# MFAP4 Deficiency Attenuates Angiotensin II-Induced Abdominal Aortic Aneurysm Formation Through Regulation of Macrophage Infiltration and Activity

**DOI:** 10.3389/fcvm.2021.764337

**Published:** 2021-11-05

**Authors:** Bartosz Pilecki, Paulo V. S. D. de Carvalho, Katrine L. Kirketerp-Møller, Anders Schlosser, Karin Kejling, Magdalena Dubik, Nicklas P. Madsen, Jane Stubbe, Pernille B. L. Hansen, Thomas L. Andersen, Jesper B. Moeller, Niels Marcussen, Vasco Azevedo, Svend Hvidsten, Christina Baun, Guo-Ping Shi, Jes S. Lindholt, Grith L. Sorensen

**Affiliations:** ^1^Department of Cancer and Inflammation Research, Institute of Molecular Medicine, University of Southern Denmark, Odense, Denmark; ^2^Department of General Biology, Institute of Biological Sciences, Federal University of Minas Gerais (UFMG), Belo Horizonte, Brazil; ^3^Department of Mathematics and Informatics, University of Southern Denmark, Odense, Denmark; ^4^Department of Cardiovascular and Renal Research, Institute of Molecular Medicine, University of Southern Denmark, Odense, Denmark; ^5^Cardiovascular, Renal and Metabolism, Innovative Medicines and Early Development Biotech Unit, AstraZeneca, Gothenburg, Sweden; ^6^Department of Pathology, Odense University Hospital, Odense, Denmark; ^7^Pathology Research Unit, Institute of Clinical Research and Institute of Molecular Medicine, University of Southern Denmark, Odense, Denmark; ^8^Danish Institute for Advanced Study, University of Southern Denmark, Odense, Denmark; ^9^Department of Nuclear Medicine, Odense University Hospital, Odense, Denmark; ^10^Department of Medicine, Brigham and Women's Hospital and Harvard Medical School, Boston, MA, United States; ^11^Department of Thoracic, Heart and Vascular Surgery, Odense University Hospital, Odense, Denmark

**Keywords:** abdominal aortic aneurysm, extracellular matrix, inflammation, macrophage, matrix metalloproteinases

## Abstract

**Objective:** Abdominal aortic aneurysm (AAA) is a common age-related vascular disease characterized by progressive weakening and dilatation of the aortic wall. Microfibrillar-associated protein 4 (MFAP4) is an extracellular matrix (ECM) protein involved in the induction of vascular remodeling. This study aimed to investigate if MFAP4 facilitates the development of AAA and characterize the underlying MFAP4-mediated mechanisms.

**Approach and Results:** Double apolipoprotein E- and *Mfap4*-deficient (*ApoE*^−/−^*Mfap4*^−/−^) and control apolipoprotein E-deficient (*ApoE*^−/−^) mice were infused subcutaneously with angiotensin II (Ang II) for 28 days. *Mfap4* expression was localized within the adventitial and medial layers and was upregulated after Ang II treatment. While Ang II-induced blood pressure increase was independent of *Mfap4* genotype, *ApoE*^−/−^*Mfap4*^−/−^ mice exhibited significantly lower AAA incidence and reduced maximal aortic diameter compared to *ApoE*^−/−^ littermates. The *ApoE*^−/−^*Mfap4*^−/−^ AAAs were further characterized by reduced macrophage infiltration, matrix metalloproteinase (MMP)-2 and MMP-9 activity, proliferative activity, collagen content, and elastic membrane disruption. MFAP4 deficiency also attenuated activation of integrin- and TGF-β-related signaling within the adventitial layer of AAA tissues. Finally, MFAP4 stimulation promoted human monocyte migration and significantly upregulated MMP-9 activity in macrophage-like THP-1 cells.

**Conclusion:** This study demonstrates that MFAP4 induces macrophage-rich inflammation, MMP activity, and maladaptive remodeling of the ECM within the vessel wall, leading to an acceleration of AAA development and progression. Collectively, our findings suggest that MFAP4 is an essential aggravator of AAA pathology that acts through regulation of monocyte influx and MMP production.

## Introduction

Abdominal aortic aneurysm (AAA) is a focal pathological dilation of the aorta associated with substantial morbidity and mortality due to the potentially fatal consequence of aortic rupture (1). AAA incidence has been observed to decline in some European populations (2), possibly due to benefits of screening programs (3, 4) or changes in population trends of cardiovascular risk factors. However, AAA mortality has not declined globally (5). Pathological mechanisms driving the formation of AAA include inflammation, smooth muscle cell (SMC) apoptosis, neovascularization, and extracellular matrix (ECM) degradation (1), which contribute to vascular remodeling and weakening of the aortic wall. The current clinical approach to treatment includes open or endovascular surgical repair when the aortic diameter has attained sufficient expansion linked to a high probability of rupture, and no validated pharmacological therapy against AAA exists (6, 7). Most AAAs of lesser diameter continue to grow and will eventually require surgical repair, highlighting a need to improve the knowledge of the mechanisms involved in development and progression of aortic aneurysms.

We have previously shown that microfibrillar-associated protein 4 (MFAP4) is an ECM protein with relatively high expression in the heart and arteries and that systemic MFAP4 levels vary with cardiovascular disease (8, 9) as well as fibrotic disease (10–12). We have demonstrated that MFAP4 binds specifically to the ECM fibrils, fibrillin, elastin, and collagen (13) and that it can activate various cells through RGD-dependent integrin ligation and downstream focal adhesion kinase (FAK)-dependent signaling (14). Unchallenged MFAP4-deficient mice exhibit mild pulmonary airspace enlargement (15) but otherwise appear healthy. However, when subjected to carotid artery ligation, *Mfap4*-deficient mice show delayed neointimal formation as well as reduced proliferation, apoptosis and inflammatory infiltration within the arterial wall (16).

Based on these observations, we hypothesized that MFAP4 might aggravate AAA formation and progression. We used a murine model of AAA development based on angiotensin II (Ang II) infusion in double apolipoprotein E- and *Mfap4*-deficient (*ApoE*^−/−^*Mfap4*^−/−^) mice and control apolipoprotein E-deficient (*ApoE*^−/−^) littermates as well as cell culture studies to establish a mechanistic role of MFAP4 in AAA pathophysiology.

## Materials and Methods

Additional details on the methods are provided in the Supplementary Material.

### Experimental Animals

*Mfap4*-deficient (*Mfap4*^−/−^) mice were generated in-house as previously described (16) and crossbred with C57BL/6N mice (Charles River Laboratories International) for >10 generations before they were used for experiments.

*ApoE*-deficient (B6.129P2-*Apoe*^tmlUnc^/J, stock nr 002052, *ApoE*^−/−^) mice were obtained from Jackson Laboratory and back-crossed to the C57BL/6N background. *ApoE*^−/−^ mice and double *ApoE-* and *Mfap4*-deficient (*ApoE*^−/−^*Mfap4*^−/−^) littermate mice were produced by *ApoE*^−/−^*Mfap4*^+/−^ breeding pairs.

The mice were housed in separate single cages during the course of the experiment. All animal experiments were approved by the National Animal Experiments Inspectorate of Denmark (permit numbers 2012-15-2934-00047 and 2015-15-0201-00474).

### Induction of AAA

Experimental AAAs were induced using a continuous infusion of Ang II as described previously (17). This model shows a strong male gender preference, recapitulating the much higher incidence of human AAA in men than in women (18). Therefore, this study only included male mice in accordance with the guidelines described in the ATVB Council Statement (19). Male *ApoE*^−/−^*Mfap4*^−/−^ and littermate *ApoE*^−/−^ control mice were fed western diet 1 week before surgery and throughout the experiment. At the age of 10–12 weeks, subcutaneous osmotic minipumps (Alzet® Model 2004, DURECT^TM^ Corporation, Cupertino, CA, USA) were installed via a mid-scapular incision under mild anesthesia (2% isoflurane, IsoFlo® vet, Orion Pharma, Nivå, Denmark) supplemented with analgesia (subcutaneous injection of 5 μg/g carprofen, Rimadyl, Pfizer, Ballerup, Denmark). Adequacy of anesthesia was monitored throughout the procedure by the toe pinch reflex.

The pumps delivered saline or Ang II (Calbiochem, Merck Millipore, Darmstadt, Germany) at 1,000 ng/kg/min for 9, 21, and 28 days. The body weight and overall well-being were regularly monitored for all mice throughout the treatment period.

Mice were euthanized by CO_2_/O_2_ asphyxiation. Abdominal aortic tissue and/or serum was sampled and snap-frozen 9 days after surgery. Aortic diameter (AD) was measured 28 days after surgery. AAA severity was scored as previously described (20). The cardiovascular system was perfused with sterile PBS and the hearts were dissected, rinsed with sterile PBS and weighed. The aortas were carefully isolated from the heart to the iliac bifurcation, cleaned from fat and connective tissue, weighed, mounted on black wax and measured. The parts of the aortas with a maximum diameter were subsequently fixed in 4% (v/w) formaldehyde for 24 h, rinsed in PBS and paraffin-embedded.

### Aortic Diameter Measurements

Maximal AAA diameter in dissected aortas from *ApoE*^−/−^ and *ApoE*^−/−^*Mfap4*^−/−^ mice was measured using a 5 mm measuring scale (Ted Pella, Inc., Redding, CA, USA) and a Canon EOS 6D camera. The measurements were performed in affected regions using Adobe® Photoshop® CC2018 (San Jose, CA, USA) in a blinded manner by two independent investigators. AAA was defined as a diameter increase >50% compared to the average aortic diameter of saline-infused mice.

### RNA *in situ* Hybridization

*In situ* hybridization was performed using a modified version of the RNAScope 2.5 high-definition procedure (Advanced Cell Diagnostics, Newark, CA, USA). Mouse aortic tissues were hybridized with 20 probe pairs (421391, Advanced Cell Diagnostics) targeting nucleotides 98-1231 of mouse Mfap4 mRNA (accession number NM_029568.2) followed by branched DNA signal amplification and tyramide enhancement visualized with Liquid Permanent Red (Agilent). The sections were subsequently immunostained with anti-α-smooth muscle actin (α-SMA) antibodies (Agilent) detected with anti-mouse BrightVision horseradish peroxidase (ImmunoLogic, Duiven, the Netherlands) and visualized with Deep Space Black (BIocare Medical, Pacheco, CA, USA).

### Immunohistochemistry

Four μm-thick serial sections were stained with hematoxylin and eosin (H&E), Verhoeff-Van Gieson, Picrosirius red and for: α-SMA, cleaved caspase-3, CD45, F4/80, Ki67, MMP-9, MMP-2, CD31, CD11b, phosphorylated (p)FAK, pSMAD2, pSMAD3, and MFAP4 (Supplementary Table 1). All immunostainings were counter-stained with hematoxylin. The stainings were performed on a Dako Autostainer Universal Staining System (Dako, Denmark A/S, Glostrup, Denmark). Stained sections were scanned at 20x magnification using NanoZoomer-XR (Hamamatsu Photonics, Hamamatsu, Japan).

### Morphometric Analysis

The scanned images were analyzed in a blinded manner using Adobe Photoshop or ImageJ.

Ki67-positive nuclei, CD31-positive microvessels, and MMP-9-positive cells were quantified as cells per section. Collagen, α-SMA, cleaved caspase-3, CD45, F4/80, CD11b, MFAP4, MMP-2, pSMAD2, pSMAD3, and pFAK stainings were quantified as staining-positive area using automated color threshold analysis. Elastic fiber degradation was assessed as a percentage of destroyed Verhoeff van Gieson-positive tissue area. Briefly, the damaged regions showing degradation of proper elastic lamellar structure were delineated and presented as a fraction of a total intima-media area. All analyses were performed in a blinded manner. Representative images of isotype control stainings are shown in Supplementary Figure 1. Representative images of entire aortic sections are shown in Supplementary Figure 2.

### Immunofluorescence

Four μm-thick serial sections were deparaffinized, subjected to antigen retrieval with citrate buffer (pH 6.0), blocked with 3% BSA and subsequently double-stained for Ki-67 and CD45 or Ki-67 and α-SMA (Supplementary Table 1). The sections were counterstained with DAPI and mounted using Fluorescent Mounting Medium (Dako). Fluorescent images were visualized and acquired using Olympus BX63 microscope (Olympus) and X-cite 120LED (Lumen Dynamics) with an Olympus DP80 camera and analyzed using ImageJ.

### Measurement of Serum MFAP4

Serum levels of mouse MFAP4 were measured using a modified AlphaLISA immunoassay (Perkin Elmer, Waltham, MA, USA) as described previously (8). The two utilized anti-MFAP4 monoclonal antibodies (HG-HYB 7-14 and HG-HYB 7-18) had been raised against human recombinant MFAP4 and cross-react with the murine MFAP4 homolog due to very high sequence similarity. Data are presented as U/ml. When measured in human serum, 1 U/ml of MFAP4 corresponds to a concentration of 38 ng/ml.

### THP-1 Cell Culture and Stimulation

THP-1 human monocyte leukemia cell line (ATCC) was grown in RPMI-1640 medium (Gibco, TermoFisher) supplemented with 10% FBS (Sigma-Aldrich), 5,000 U/ml penicillin, 5,000 μg/ml streptomycin, and 200 mM L-glutamine (all from Gibco) at 37°C and 5% CO_2_ humidity. The cells were subcultured every second-third day.

MaxiSorp 96-well plates were coated overnight at 4°C with human serum albumin (HSA) or immobilized MFAP4 (both 10 μg/ml). The cells seeded (40.000 cells/well) and differentiated with 5 nM phorbol 12-myristate 13-acetate (PMA; Sigma) for 48 h, equilibrated with complete medium for 24 h, and serum-starved for 48 h. The cells were then stimulated with 20 ng/ml TNF (R&D Systems) for 48 h. Zymography on culture supernatants was performed essentially as described above. Cell proliferation rate was assessed using WST-1 assay (Sigma) according to the manufacturer's instructions. Cell viability was assessed by CytoTox-ONE^TM^ Homogeneous Membrane Integrity Assay (Promega) according to the manufacturer's instructions. The zymography results were normalized to the cell proliferation index.

### SMC Culture and Stimulation

Fetal human primary aortic SMCs (Cell Applications) were grown in Smooth Muscle Cell Growth Medium (Cell Applications) supplemented with 5,000 U/ml penicillin and 5,000 μg/ml streptomycin. Cells from passages 3–10 were used.

The cells were seeded at HSA- and MFAP4-coated plates essentially as described above (16.800 cells/well), starved overnight in Smooth Muscle Cell Basal Medium (Cell Applications) and stimulated with indicated concentrations of TNF or Ang II for 24 h. Zymography on culture supernatants was performed essentially as described above.

### Human Monocyte Isolation

Human peripheral blood mononuclear cells were isolated from buffy coats obtained from the local blood bank (permit nr DP086) using Ficoll-Paque Plus density gradient centrifugation (GE Healthcare). Briefly, the samples were layered over 15 ml of Ficoll and centrifuged brake-free for 25 min at 800 g at room temperature. The interface was removed and washed twice with PBS containing 2% FBS and 1 mM EDTA. The cells were resuspended in PBS containing 2% FBS and 1 mM EDTA. The monocytes were enriched using the EasySep™ Human CD14 Positive Selection Kit (Stemcell Technologies) according to the manufacturer's instructions. The purity of isolated monocytes was tested by flow cytometry immediately after isolation by staining with anti-CD14-FITC antibody (BD Biosciences) and was >97% in all experiments.

### Monocyte Migration Assay

The lower sides of the Transwell inserts with 5.0 μm pores (Corning) were coated with 10 μg/cm^2^ MFAP4 or HSA overnight at 4°C, washed with PBS, blocked with 10 mg/ml HSA for 1 h at room temperature and washed again. The monocytes were seeded in the upper chamber (100.000 cells/insert) in serum-free RPMI medium containing 0.5% FBS. In some experiments, the cells were pre-incubated with anti-integrin α_V_β_3_, anti-integrin α_V_β_5_ (both from Merck Millipore), or isotype control antibody (Thermo Fisher) for 30 min at room temperature before seeding. The lower chamber contained serum-free RPMI medium with 0.5% FBS ± 100 ng/ml human recombinant CCL-2 (R&D Systems). The cells were allowed to migrate for 3 h, after which the upper sides of the filters were washed with PBS and swiped with a cotton swab to remove any non-migrated cells. The lower sides of the filters were then stained with Hemacolor (Sigma) and divided into four fields. The migrated cells in each field were counted in a blinded manner by two independent investigators.

### Statistical Analysis

The normality of data was assessed using Shapiro-Wilk test. Levene's test was used to assess equality of variances. Non-normally distributed data were analyzed using Mann-Whitney *U*-test. Normally distributed data were analyzed using one-way ANOVA or Student's *t*-test. Comparisons of aneurysm incidence were performed using Fisher's exact test. Data are presented as means + SEM unless otherwise stated. Significance was accepted if *p* < 0.05. All statistical analyses were performed using GraphPad Prism (GraphPad.com).

## Results

### MFAP4 Expression in Normal Aorta and AAA

**Figure 1:**
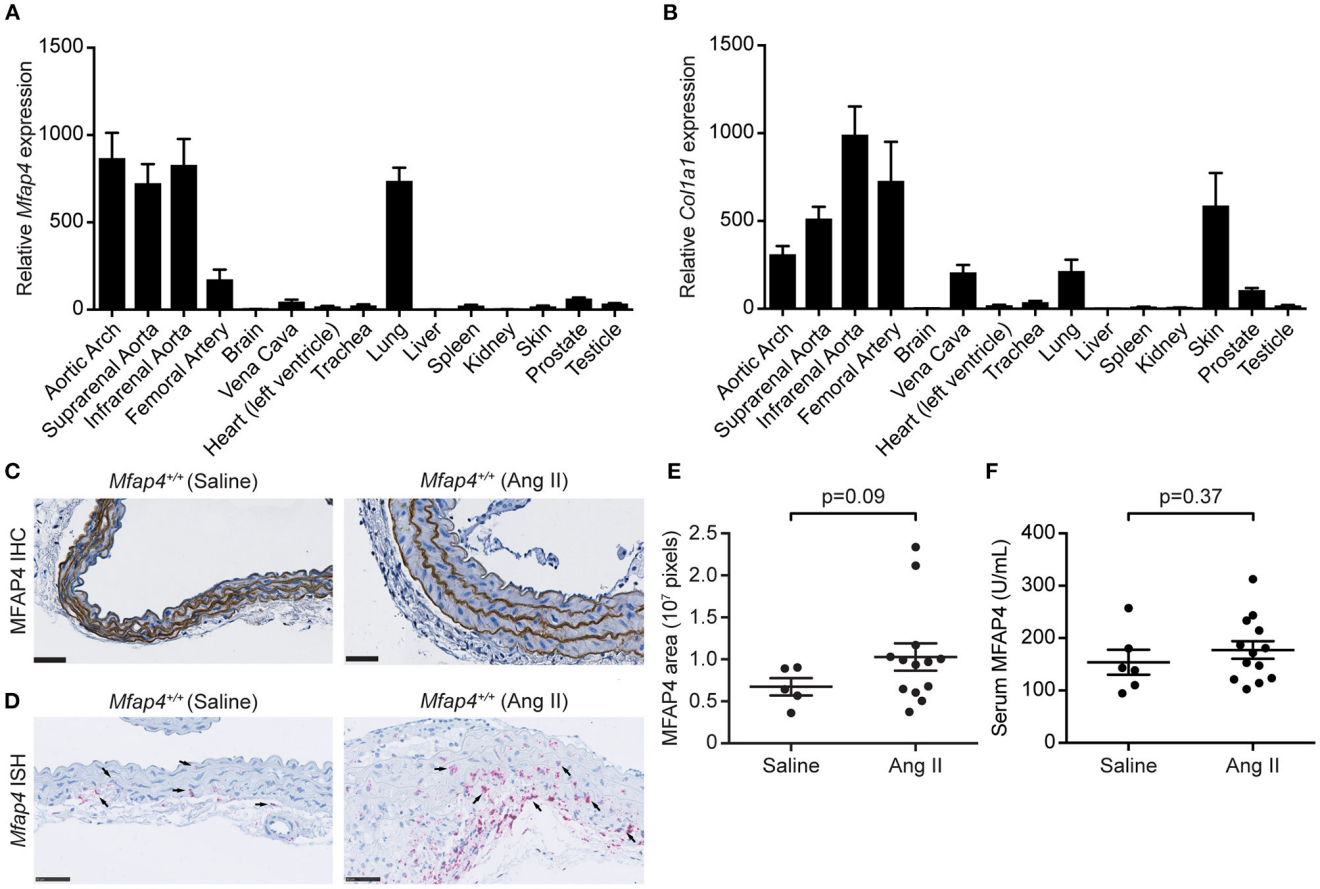
MFAP4 expression in normal aorta and AAA tissue. **(A,B)** Quantitative real-time PCR analysis of **(A)** *Mfap4* and **(B)** *Col1a1* mRNA transcripts in C57BL/6N mouse tissues (*n* = 7). Expression levels are normalized against eukaryotic 18S rRNA and presented as means + SEM. **(C,D)** Representative images of **(C)** MFAP4 immunohistochemical staining (IHC) and **(D)** *Mfap4 in situ* hybridization staining (ISH) in abdominal aortic sections in saline-infused and Ang II-infused *ApoE*^−/−^ (*Mfap4*^+/+^) mice. Black arrows indicate examples of positive cells. *n* = 5–13. Scale bar = 50 μm. **(E)** Semi-quantitation of MFAP4 IHC staining intensity in the aortas of saline- and Ang II-infused *ApoE*^−/−^ mice. **(F)** MFAP4 concentration in serum from saline- and Ang II-infused *ApoE*^−/−^ mice. Data are analyzed with Mann-Whitney *U*-test.

Initially, we performed RT-qPCR analysis to assess the relative *Mfap4* mRNA levels in the aorta as well as 19 additional tissues from wild-type C57BL/6N mice. The relative expression levels of *Mfap4* were highest in the lung and arteries (Figure 1A). MFAP4 is an ECM molecule with the capacity to bind various ECM fibers including collagen (21). In line with this, the tissue expression profile of type I collagen (*Col1a1*) mRNA resembled the *Mfap4* expression profile (Figure 1B).

In agreement with previous observations (16), MFAP4 was predominantly localized to the arterial elastic fibers by immunohistochemistry, and this localization was unaltered after 28 days of Ang II infusion (Figure 1C). To identify *Mfap4*-expressing cells, we performed *in situ* hybridization staining for *Mfap4* transcript that revealed that *Mfap4* mRNA is expressed in adventitial cells (presumably adventitial fibroblasts) and medial SMCs and that it is upregulated upon AAA infusion (Figure 1D and Supplementary Figure 3). Ang II-induced AAA development did not significantly impact MFAP4 deposition within the aortic ECM (Figure 1E) or circulating serum MFAP4 levels (Figure 1F).

### *Mfap4* Deficiency Does Not Affect HR, MAP, and Heart Weight Development After Ang II Infusion

Following, we assessed the changes in basic physiological parameters after Ang II infusion. We have previously reported that MAP is not affected by *Mfap4* ablation in unchallenged mice when measured over periods of minutes-to-hours 5 days after placing indwelling catheters (16). In the present study, HR and MAP were measured continuously for 7 days using indwelling catheters in conscious *ApoE*^−/−^ and *ApoE*^−/−^*Mfap4*^−/−^ mice infused with Ang II. A significantly increased HR and a tendency for increased MAP was observed in the active night period (6 p.m. to 6 a.m.) compared to the day period (6 a.m. to 6 p.m.) for both *ApoE*^−/−^ and *ApoE*^−/−^*Mfap4*^−/−^ mice after Ang II treatment. However, we did not observe any significant differences in either HR or MAP between Ang II-infused *ApoE*^−/−^ and *ApoE*^−/−^*Mfap4*^−/−^ mice (Supplementary Figures 4A,B). Likewise, heart weight and heart-to-body weight ratio were significantly increased after Ang II infusion but not influenced by *Mfap4* genotype (Supplementary Figures 4C,D).

### *Mfap4* Deficiency Attenuates Ang II-Induced AAA Development

**Figure 2:**
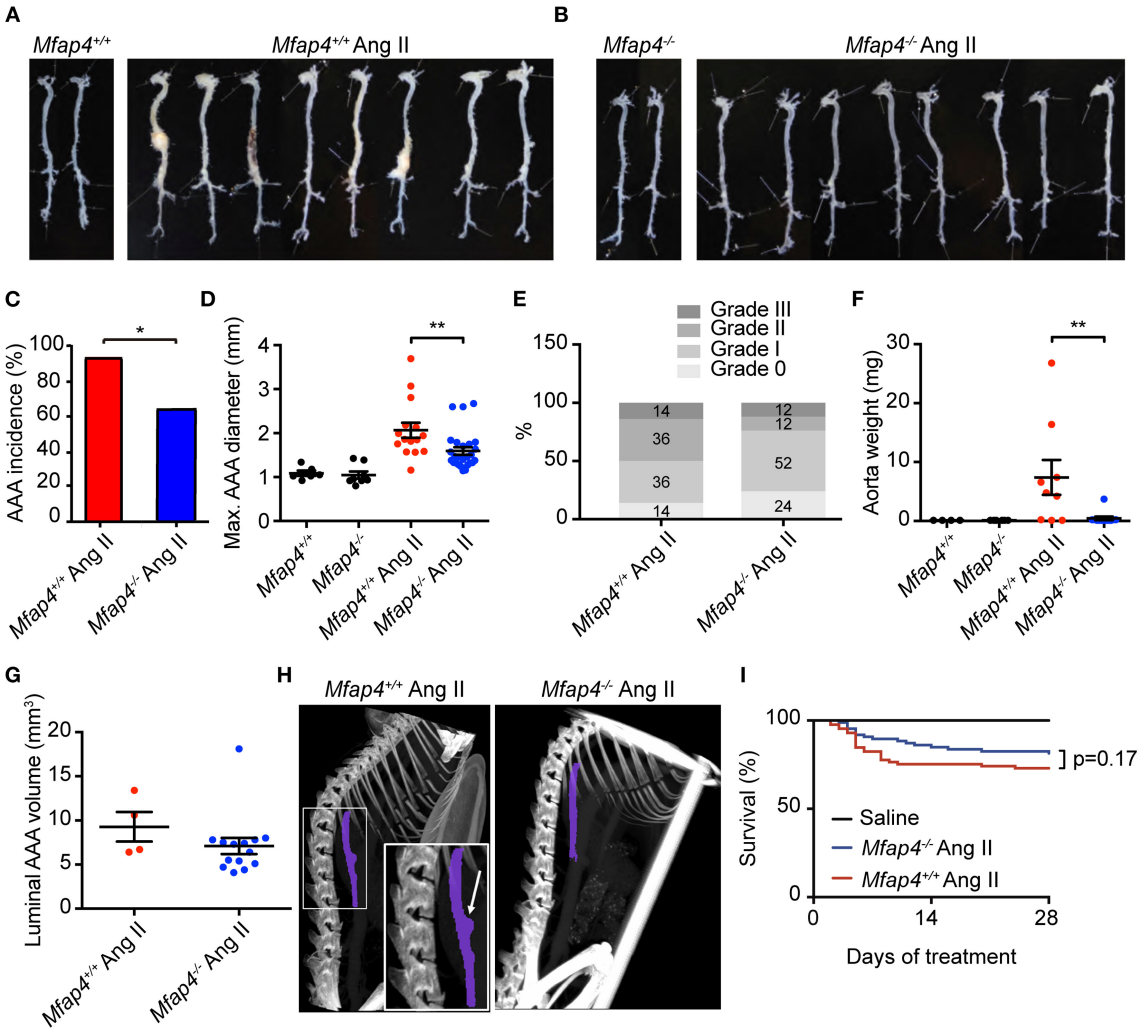
*Mfap4* deficiency attenuates Ang II-induced AAA development. **(A,B)** Representative photographs of dissected aortic segments from the aortic arch to the iliac bifurcation from **(A)** *ApoE*^−/−^ (*Mfap4*^+/+^) and **(B)** *ApoE*^−/−^*Mfap4*^−/−^ (*Mfap4*^−/−^) mice infused with saline (left panels) or Ang II (right panels) for 28 days. **(C)** AAA incidence. *n* = 15 (*Mfap4*^+/+^ Ang II), 26 (*Mfap4*^−/−^ Ang II). **(D)** Quantification of maximal AAA diameter. *n* = 6–8 (saline), 15–26 (Ang II). **(E)** AAA severity scoring. *n* = 14 (*Mfap4*^+/+^ Ang II), 25 (*Mfap4*^−/−^ Ang II). **(F)** Quantification of dissected aorta weight. *n* = 4–6 (saline), 9–12 (Ang II). **(G)** Quantification of luminal AAA volume assessed by micro-CT. *n* = 4 (*Mfap4*^+/+^ Ang II), 14 (*Mfap4*^−/−^ Ang II). **(H)** Representative micro-CT images of the vascular luminal volume spanning a distance of 4 vertebrae after 28 days of Ang II infusion. Purple color demarks the investigated region of interest. The arrow indicates an aortic AAA. **(I)** Survival analysis. *p < 0.05, **p < 0.01, analyzed with Fisher's exact test **(C)** or Mann-Whitney *U*-test **(D–F)**.

We next asked whether MFAP4 plays a role in the development of Ang II-induced AAA. As expected, none of the saline-infused mice developed AAAs. In contrast, 28 day-long Ang II infusion caused suprarenal AAA development, with *ApoE*^−/−^ *Mfap4*^−/−^ mice showing a significantly lower incidence rate (62%) compared to 93% incidence rate in *ApoE*^−/−^ mice (Figures 2A–C). Moreover, Ang II-infused *ApoE*^−/−^*Mfap4*^−/−^ mice exhibited significantly lower maximal outer AAA diameter compared to *ApoE*^−/−^ *mice* (Figure 2D) as well as ameliorated AAA severity (Figure 2E). The dissected aorta weight was significantly reduced from 7.4 ± 3.0 mg in Ang II-treated *ApoE*^−/−^ mice to 0.4 ± 0.3 mg in *ApoE*^−/−^*Mfap4*^−/−^ mice (Figure 2F). A similar trend was observed in the vascular luminal volume between *ApoE*^−/−^ and *ApoE*^−/−^*Mfap4*^−/−^ mice when measured over a distance of four specific vertebrae using micro-CT in a limited number of samples (Figures 2G,H). On the other hand, there was no significant difference in survival (caused by early aneurysm rupture) between *ApoE*^−/−^ and *ApoE*^−/−^*Mfap4*^−/−^ mice (Figure 2I). In addition, no effect of Ang II infusion or MFAP4 deficiency was observed on serum total cholesterol or triglyceride levels (Supplementary Figure 5).

### *Mfap4* Deficiency Reduces Macrophage Infiltration, MMP Activity, and FAK Activation in Ang II-Induced AAAs

**Figure 3:**
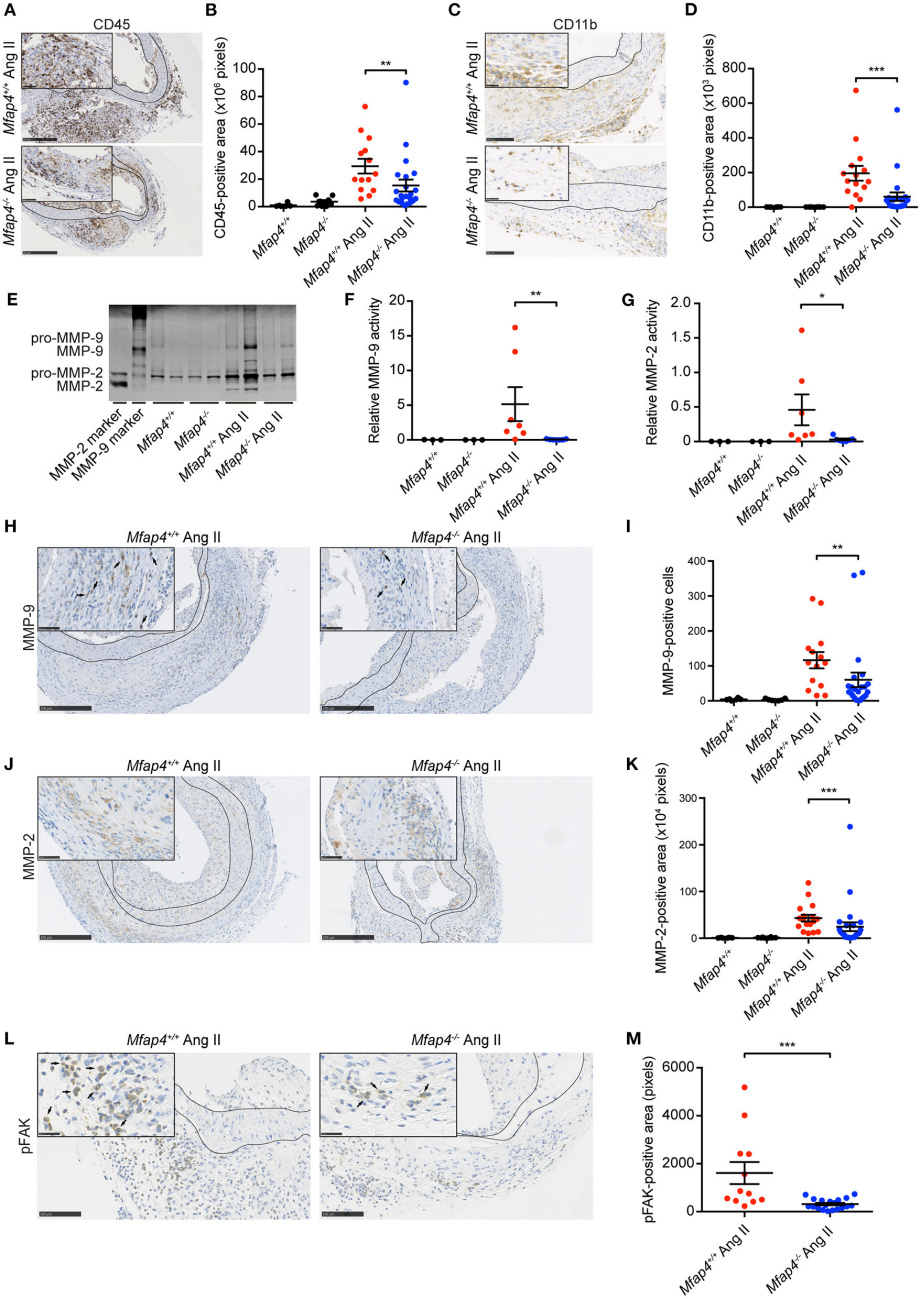
*Mfap4* deficiency reduces macrophage infiltration, MMP activity, and FAK activation in Ang II-induced AAAs. **(A–D)** Morphometric analysis of **(A,B)** CD45 and **(C,D)** CD11b stainings of aortic sections of *ApoE*^−/−^ (*Mfap4*^+/+^) and *ApoE*^−/−^*Mfap4*^−/−^ (*Mfap4*^−/−^) mice after 28 days of Ang II infusion. *n* = 6–8 (saline), 14–24 (Ang II). **(E–G)** Representative MMP zymogram **(E)** of aortic lysates from saline- and Ang II-infused mice after 9 days with corresponding densitometric quantification of **(F)** MMP-9 and **(G)** MMP-2 activity. *n* = 3–7. **(H–M)** Morphometric analysis of **(H,I)** MMP-9, **(J,K)** MMP-2, and **(L,M)** phosphorylated FAK (pFAK) stainings of aortic sections of *ApoE*^−/−^ (*Mfap4*^+/+^) and *ApoE*^−/−^*Mfap4*^−/−^ (*Mfap4*^−/−^) mice after 28 days of Ang II infusion. Black arrows indicate examples of positive cells. Black lines delineate borders between intimal, medial and adventitial layers. *n* = 6–8 (saline), 12–26 (Ang II). Representative pictures are shown. Scale bar = 250 μm/50 μm **(A,H,J)**, 100 μm/25 μm **(B,L)**. *p < 0.05, **p < 0.01, ***p < 0.001, analyzed with Mann-Whitney *U*-test.

Following, we evaluated the MFAP4-dependent changes in the inflammatory responses within the aortic wall. Ang II-induced inflammatory infiltration, quantified as the CD45-positive area, was significantly attenuated within the aortas of *ApoE*^−/−^*Mfap4*^−/−^ mice compared to *ApoE*^−/−^ littermates (Figures 3A,B). Moreover, Ang II-infused *ApoE*^−/−^*Mfap4*^−/−^ mice showed a potent reduction in CD11b-positive area in the aortas of Ang II-infused *ApoE*^−/−^*Mfap4*^−/−^ mice compared to *ApoE*^−/−^ littermates (Figures 3C,D), suggesting monocytes/macrophages to be the affected leukocyte type. We confirmed this by staining for another macrophage marker F4/80, which yielded comparable results (data not shown). Furthermore, CD11b-positive area analyzed exclusively within the adventitial layer was also significantly lowered in Ang II-infused *ApoE*^−/−^*Mfap4*^−/−^ mice compared to *ApoE*^−/−^ littermates (Supplementary Figure 6).

We further analyzed aortic tissue lysates (collected at day 9) by zymography to investigate MMP activity in Ang II-infused mice (Figure 3E). We observed a significant decrease in both MMP-2 (Figure 3F) and MMP-9 (Figure 3G) activity in Ang II-infused *ApoE*^−/−^*Mfap4*^−/−^ mice compared to *ApoE*^−/−^ littermates. To confirm that, we analyzed MMP protein expression in aortic sections and found that both MMP-2 and MMP-9 expression, localized predominantly in the adventitial layer, were significantly decreased in Ang II-infused *ApoE*^−/−^*Mfap4*^−/−^ mice compared to *ApoE*^−/−^ littermates (Figures 3H–K). These results suggest that MFAP4 promotes inflammatory responses in macrophages during AAA development.

As integrin receptors serve as main MFAP4 cellular ligands, we stained AAA sections for pFAK as a proxy for activation of integrin signaling pathways. We observed that total pFAK-positive adventitial area as well as pFAK-positive area normalized to adventitial area were significantly reduced in Ang II-infused *ApoE*^−/−^*Mfap4*^−/−^ mice compared to *ApoE*^−/−^ littermates (Figures 3L,M and not shown).

### *Mfap4* Deficiency Reduces Cellular Proliferation, Apoptosis, and Microvessel Number in Ang II-Induced AAAs

**Figure 4:**
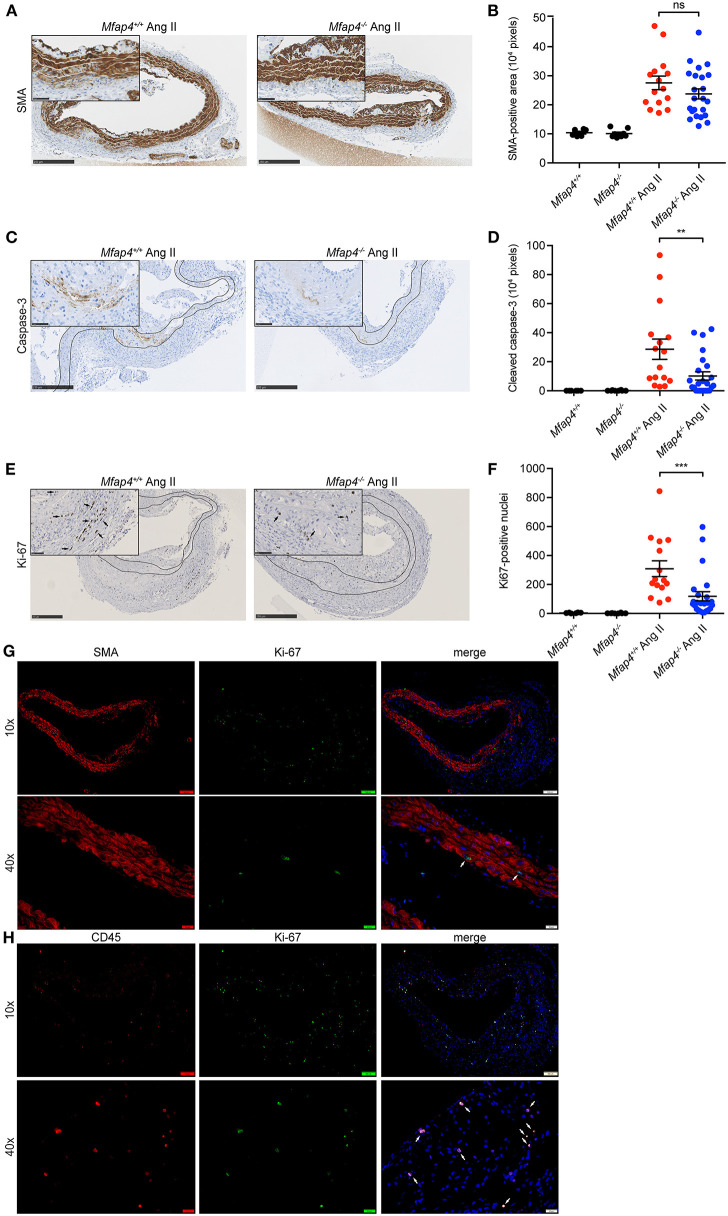
*Mfap4* deficiency reduces cellular proliferation and apoptosis in Ang II-induced AAAs. Morphometric analysis of **(A,B)** α-smooth muscle actin (SMA), **(C,D)** cleaved caspase-3, and **(E,F)** Ki-67 stainings of aortic sections of *ApoE*^−/−^ (*Mfap4*^+/+^) and *ApoE*^−/−^*Mfap4*^−/−^ (*Mfap4*^−/−^) mice after 28 days of Ang II infusion. Black arrows indicate examples of positive cells. Black lines delineate borders between intimal, medial and adventitial layers. **(G,H)** Double immunofluorescent staining of Ki-67 and SMA **(G)** or CD45 **(H)**. White arrows indicate examples of double-positive cells. *n* = 6–8 (saline), 15–26 (Ang II). Representative pictures are shown. Scale bar = 250 μm/50 μm **(A,C,E)**, 100 μm/20 μm **(G,H)**. **p < 0.01, ***p < 0.001, analyzed with Mann-Whitney *U*-test. ns, non-significant.

Medial α-SMA-positive area remained unchanged between Ang II-infused *ApoE*^−/−^ and *ApoE*^−/−^*Mfap4*^−/−^ mice (Figures 4A,B).

We then investigated the degree of cellular apoptosis and proliferation within the vessel wall. Ang II infusion resulted in an overall increase in both cleaved caspase 3-positive area and Ki-67 positive cell number. Apart from few isolated cells found throughout the tissue, the majority of cleaved caspase 3-positive staining was localized within the media at the sites of SMC degeneration. Ang II-induced apoptosis was significantly attenuated in *ApoE*^−/−^*Mfap4*^−/−^ mice compared to *ApoE*^−/−^ littermates (Figures 4C,D).

On the other hand, Ki-67-positive cells were found throughout the vessel wall but predominantly in the adventitial layer. Ki-67 positive cell number was significantly reduced in Ang II-infused *ApoE*^−/−^*Mfap4*^−/−^ mice compared to *ApoE*^−/−^ littermates (Figures 4E,F). Moreover, double immunofluorescent staining revealed that while single SMA-positive medial SMCs stained positive for Ki-67 (Figure 4G), the vast majority of Ki67-positive cells were CD45-positive infiltrating leukocytes (Figure 4H).

CD31-positive microvessels were essentially undetectable in aortic tissues from control mice, while numerous capillary vessels were observed in Ang II-infused aortas after 28 days. Ang II-infused *ApoE*^−/−^*Mfap4*^−/−^ mice exhibited a 78% reduction in the observed number of microvessels compared to *ApoE*^−/−^ mice (Supplementary Figure 7).

### *Mfap4* Deficiency Limits Elastic Membrane Disruption as Well as Collagen Deposition and Associated Fibrotic Signaling in Ang II-Induced AAAs

**Figure 5:**
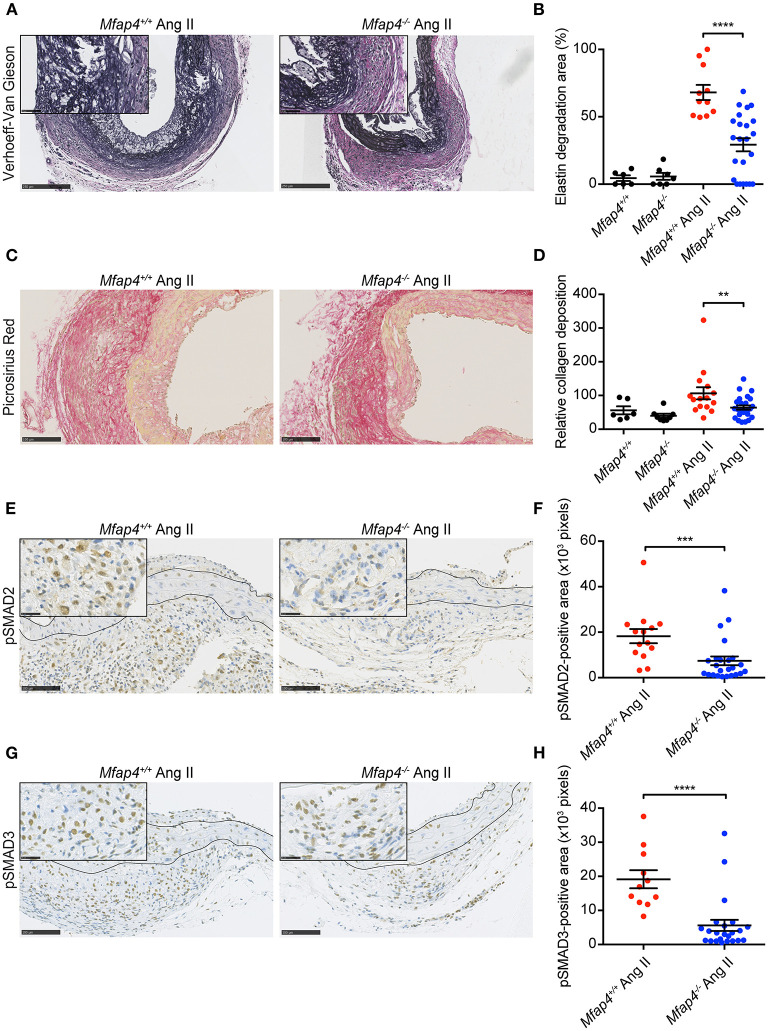
*Mfap4* deficiency limits elastic membrane disruption, collagen deposition and fibrotic signaling in Ang II-induced AAAs. Morphometric analysis of **(A,B)** elastin (Verhoeff-Van Gieson) staining, **(C,D)** collagen (Picrosirius Red) staining, **(E,F)** phosphorylated SMAD2 (pSMAD2) staining and **(G,H)** phosphorylated SMAD3 (pSMAD3) staining of aortic sections of *ApoE*^−/−^ (*Mfap4*^+/+^) and *ApoE*^−/−^*Mfap4*^−/−^ (*Mfap4*^−/−^) mice after 28 days of Ang II infusion. Black lines delineate borders between intimal, medial and adventitial layers. *n* = 6–8 (saline), 11–26 (Ang II). Representative pictures are shown. Scale bar = 250 μm/50 μm **(A)**, 100 μm **(C)**, 100 μm/25 μm **(E,G)**. **p < 0.01, ***p < 0.001, ****p < 0.0001, analyzed with Mann-Whitney *U*-test.

Ang II infusion resulted in elastic membrane disruption that was significantly attenuated in *ApoE*^−/−^*Mfap4*^−/−^ mice compared to *ApoE*^−/−^ littermates (Figures 5A,B).

We also analyzed AAA-linked fibrotic changes within the vessel wall. Ang II treatment induced adventitial collagen deposition that was significantly decreased in Ang II-infused *ApoE*^−/−^*Mfap4*^−/−^ mice compared to *ApoE*^−/−^ littermates (Figures 5C,D). To investigate the related mechanisms, we stained AAA sections for pSMAD2 and pSMAD3, key mediators of pro-fibrotic TGF-β signaling. While the medial pSMAD-positive area was modestly influenced by MFAP4 genotype (Supplementary Figure 8), both the pSMAD2- and pSMAD3-positive area in the adventitia were highly reduced in Ang II-infused *ApoE*^−/−^*Mfap4*^−/−^ mice compared to *ApoE*^−/−^ littermates (Figures 5E–H), showing that activation of TGF-β-dependent downstream signaling is significantly attenuated by MFAP4 deficiency.

### MFAP4 Promotes Monocyte Chemotaxis

**Figure 6:**
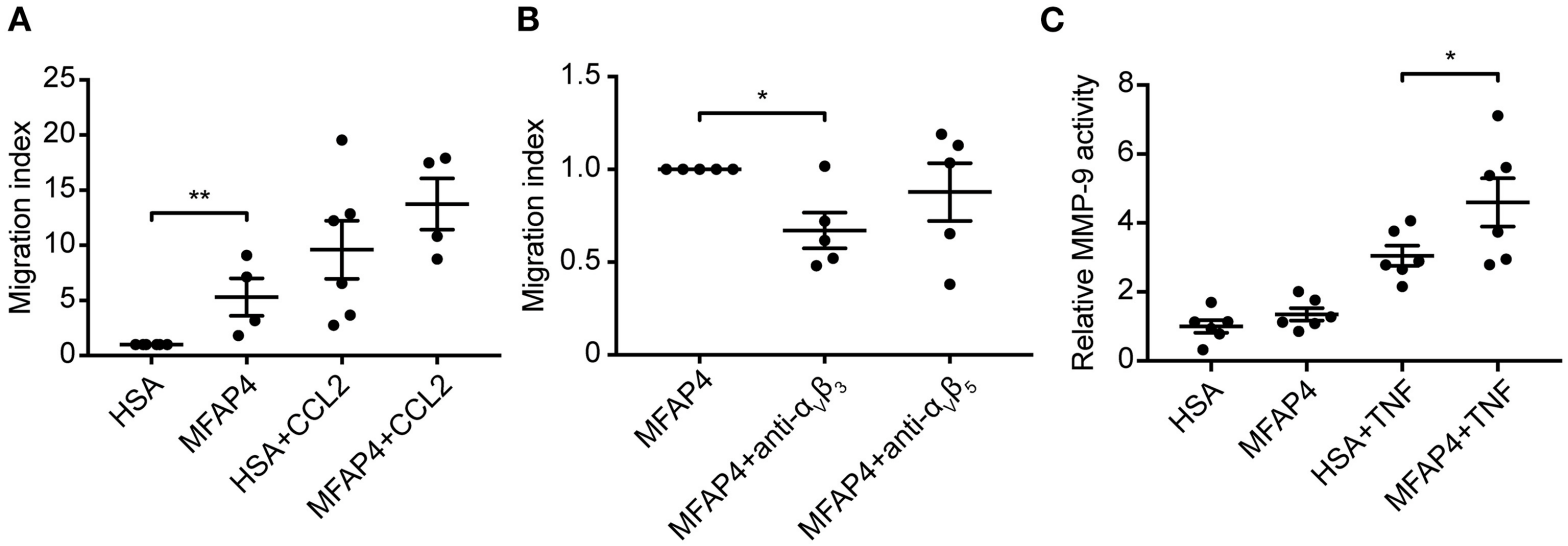
MFAP4 induces monocyte migration and MMP-9 activity in macrophage-like cells *in vitro*. **(A)** MFAP4 acts as a haptoattractant for human blood monocytes. **(B)** MFAP4-dependent monocyte directional migration can be inhibited by anti-integrin α_V_β_3_ antibody. **(C)** MFAP4 stimulation potentiates TNF-induced MMP-9 activity in differentiated THP-1 macrophage-like cells. *n* = 4–6 independent experiments. *p < 0.05, **p < 0.01, analyzed with paired *t*-test and repeated measures ANOVA followed by Bonferroni's test.

To confirm our *in vivo* findings and better understand the molecular mechanisms behind MFAP4-mediated regulation of inflammatory infiltration, we evaluated the role of MFAP4 in chemotaxis of blood monocytes. We observed that MFAP4 alone was able to stimulate directional monocyte migration and that this increase could be inhibited by blocking integrin α_V_β_3_ but not integrin α_V_β_5_ (Figures 6A,B). We observed a similar tendency for monocyte chemotaxis toward CCL-2, although it did not reach statistical significance (Figures 6A,B).

### MFAP4 Stimulation Induces MMP-9 Activity in Macrophage-Like Cells *in vitro*

Finally, we investigated if MFAP4 has a direct effect on MMP production in SMCs and macrophage-like cells. We stimulated fetal aortic SMCs and PMA-differentiated THP-1 cells with immobilized MFAP4 with or without TNF co-stimulation. MMP-2 activity in fetal aortic SMCs was independent of MFAP4 regardless of TNF or Ang II stimulation (Supplementary Figure 9A). Conversely, we observed that while TNF stimulation resulted in an overall increase in MMP-9 activity in PMA-differentiated THP-1 cells, co-stimulation with MFAP4 significantly potentiated MMP-9 activity when compared to TNF stimulation alone (Figure 6C). THP-1 cell proliferation or viability after TNF stimulation were not significantly influenced by MFAP4 (Supplementary Figures 9B,C).

## Discussion

**Figure 7:**
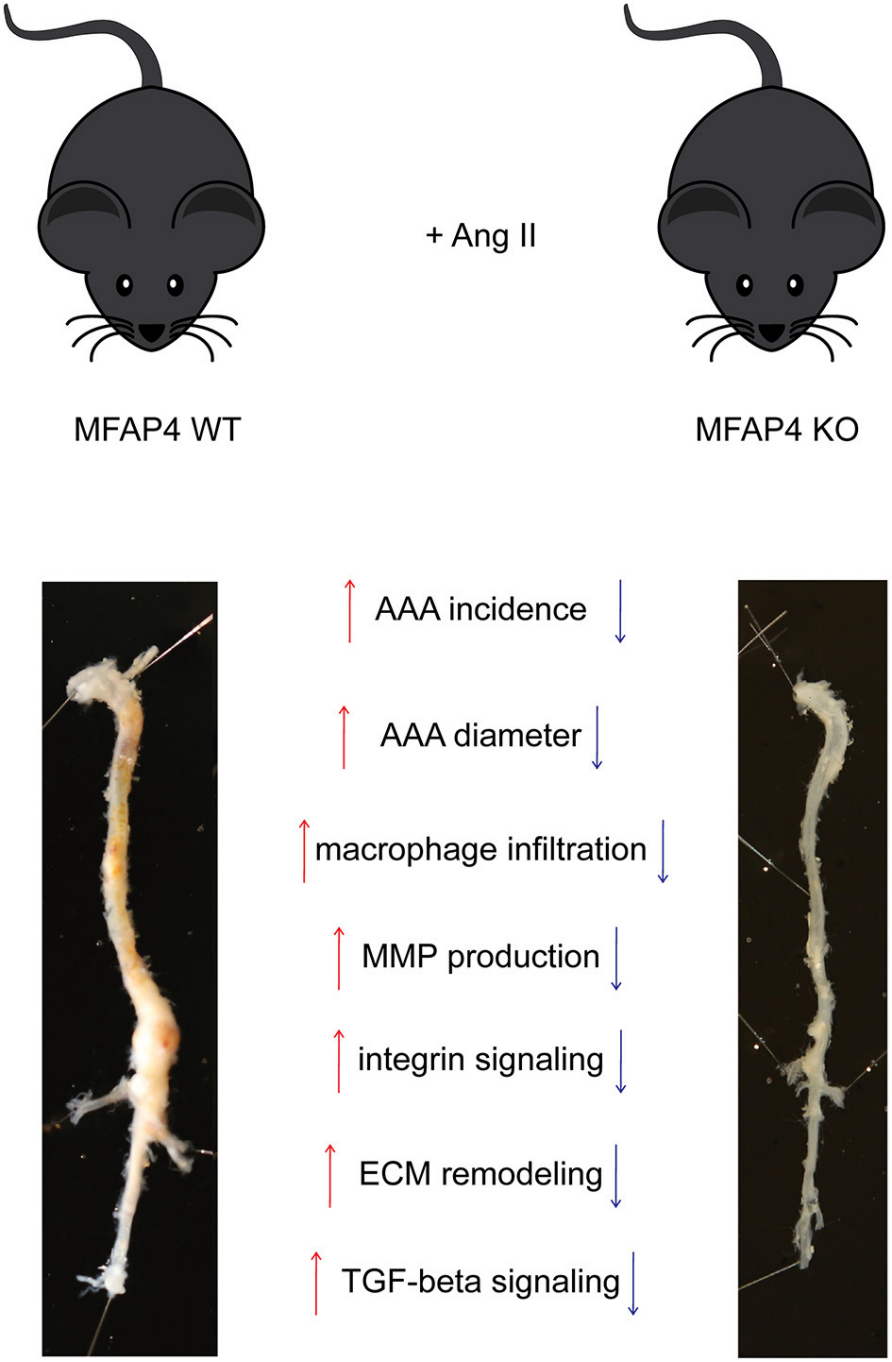
MFAP4 deficiency protects against Ang II-induced AAA pathology. Overview of the identified actions of MFAP4 in Ang II-induced AAA.

In the present study, we evaluated the role of MFAP4 in AAA pathology in mice. We showed that MFAP4 is abundantly expressed in the arteries and that its mRNA expression is upregulated after Ang II infusion. Furthermore, we demonstrated that Ang II-induced AAA formation is attenuated in *Mfap4*-deficient mice due to reduced macrophage infiltration, MMP activity, integrin signaling and vascular remodeling. We also showed that MFAP4 directly induces monocyte migration and MMP-9 activity. Thus, MFAP4 contributes to the weakening of the aortic wall and aggravates vascular pathology in an Ang II-driven model of AAA (Figure 7).

MFAP4 is a structural and functional component of elastic fibers throughout the body, abundantly present within the vascular ECM. Such expression pattern sets MFAP4 apart from the “matricellular” proteins, described to be non-structural, cell-activating ECM proteins that are virtually absent during homeostasis but show a dynamic upregulation during vascular pathogenesis (22). The present study supports a permissive role for MFAP4 in the induction of pathological vascular remodeling, previously established for outward arterial remodeling in neointima formation (16). The eliciting factors in MFAP4-mediated AAA progression may be the disease-related upregulation of integrin expression as well as concomitant growth factor signaling, known to potentiate the integrin-dependent cellular responses (23). Indeed, RGD-dependent integrin expression is induced and correlates to the degree of vascular inflammation in the Ang II-induced AAA model (24), suggesting it to be a primary driver of MFAP4-mediated effects.

MFAP4 has previously been localized to the vessel wall, but the exact aortic cell types responsible for its production and deposition have not been investigated in detail. By using *in situ* hybridization, we showed that MFAP4 transcript expression localized predominantly to adventitial cells (possibly fibroblasts) and to a lesser extent also to medial cells (vascular SMCs). Our findings are in line with recent single cell RNAseq data, where MFAP4 was found to be expressed mainly in various fibroblast subpopulations and only to a minor degree by mural cells such as vascular SMCs and pericytes (25, 26). Further investigations are needed to confirm the expression of MFAP4 in adventitial fibroblasts and identify the specific fibroblast subset responsible for MFAP4 upregulation in AAA.

The hallmark pathology of AAA is the destruction of elastic lamellae within the aortic media, associated with vessel expansion. While multiple MMPs have been reported as necessary in this aspect of AAA formation, MMP-2 and MMP-9 play a particularly important role (17), and the absence of either MMP-2 and MMP-9 is associated with lower incidence of experimental AAAs (27). In the present study, we showed that MFAP4 deficiency significantly decreased both MMP-2 and MMP-9 expression and activity. These MFAP4-dependent differences appeared to be of sufficient magnitude to infer significant changes in elastin integrity.

The cellular sources of MMPs include mesenchymal cells as well as macrophages and other leukocytes, with MMP-2 and MMP-9 predominantly derived from vascular SMCs and macrophages, respectively (28–30). Importantly, integrin α_V_β_3_ has been reported to promote cellular production of MMPs (31). We found that MFAP4 effectively stimulated MMP-9 activity in differentiated THP-1 cells *in vitro*. Conversely, vascular SMC production of MMP-2 seemed to be independent of MFAP4. While the observed effects might be cell type-specific and depend e.g., on integrin abundance and activation status, they imply an essential role for MFAP4 in driving MMP-9 production in macrophages.

MFAP4 engagement in MMP synthesis has previously been reported in human skin, where MFAP4 appeared to protect collagen integrity by reducing MMP-12 activity after UV light exposure (32). Moreover, MFAP4 is known to accelerate tropoelastin assembly into elastic fibers (13), and *Mfap4*-deficient mice develop a mild age-induced airspace enlargement linked to loss of alveolar surface (15), both indicating that MFAP4 contributes to ECM stability. These previous observations suggest that the role of MFAP4 may change from maintenance of proper tissue architecture during normal homeostatic conditions toward promotion of inflammation and remodeling in pathological settings.

Monocytes and macrophages play a critical role in vascular injury. Macrophages, arising mainly from circulating monocytes, constitute a major inflammatory cell type within AAA lesions (33). Monocyte adhesion, migration, and MMP-9 production are all increased in AAA patients and lead to aneurysm expansion (34). Here we demonstrated that Ang II-driven macrophage recruitment was significantly limited in *Mfap4*-deficient mice and that MFAP4 directly promoted haptotactic migration of monocytes via integrin α_V_β_3_ ligation. In line with that, integrin α_V_β_3_ blockade has been previously reported to attenuate monocyte/macrophage infiltration both *in vitro* and within the vessel wall (35, 36). Further supporting our observations, the crucial mediator of integrin signaling FAK has been shown to stimulate macrophage motility and MMP synthesis in experimental AAA. Importantly, activated FAK has been localized predominantly to adventitial macrophages and only rarely to medial SMCs (37). In agreement with that, we found that MFAP4 promotes FAK activation specifically in the adventitia. Taken together, these findings strongly suggest that MFAP4-integrin interaction and subsequent downstream FAK signaling promote monocyte/macrophage recruitment and activation.

Circulatory MFAP4 has been previously associated with fibrotic deposition and cirrhosis in hepatitis C as well as other conditions leading to fibrogenesis of the liver (10, 11). Moreover, direct induction of collagen synthesis in white blood cells after treatment with MFAP4 has been demonstrated (38). Together, these observations imply that MFAP4 may directly affect collagen synthesis. In agreement with that, we observed the MFAP4-dependent increase in adventitial pSMAD staining in Ang II-induced AAA. Phosphorylation of SMAD2 and SMAD3 is a key step in pro-fibrotic signaling leading to collagen deposition (39). Indeed, MFAP4 deficiency has been shown to attenuate kidney and cardiac fibrosis (40, 41), further underlining MFAP4 involvement in fibrotic tissue remodeling. Importantly, upregulation of pSMADs and other crucial components of the TGF-β signaling pathway has been reported in AAA patient samples (42). As TGF-β signaling is mostly suggested to exert a protective role in AAA pathology (43), this dysregulated, exaggerated response might be a compensatory mechanism to the pathological changes happening in the aortic wall.

MFAP4 is a ligand for integrins α_V_β_3_ and α_V_β_5_, known inducers of neovascularization (44). Angiogenesis has been associated with the risk of AAA rupture and complications (45) and is suggested to result mainly from the growth factor signaling inferred by inflammatory cells accumulating within the vessel wall (46). Therefore, the observed reduction of aortic microvessel number in *Mfap4*-deficient animals was expected. However, we did not further investigate the mechanistic role of MFAP4 in angiogenesis in this study.

Several studies have supported that clinical MFAP4 levels may be influenced by the presence of vascular aneurysms. However, the pattern of MFAP4 regulation has shown inconsistency (47–49), and a role of MFAP4 in clinical AAA remains unknown. We have recently shown that high plasma MFAP4 is associated with reduced risk of undergoing later surgical repair in AAA (50); however, this observation needs validation in an independent cohort.

An imbalance of the renin-angiotensin system has been associated with the pathogenesis of AAA (51), and Ang II-induced AAA formation in *ApoE*^−/−^ mice shares many characteristic features of the human disease, including chemokine generation, macrophage infiltration, and neovascularization (18). However, weaknesses of our study include that we only used a single model of AAA formation and thus cannot rule out whether the observed MFAP4-mediated effects are exclusively dependent on Ang II treatment. Particularly, Ang II infusion also results in development of atherosclerosis, and the importance of MFAP4 in atherosclerosis-independent AAA model remains to be investigated. On the other hand, as significant atherosclerotic lesions are first observed beyond 28 days of Ang II treatment (52), and the AAA lesions can be induced (although with lowered incidence) also in normolipidemic mice (53), it seems that atherosclerosis might develop independently of AAA. Also, we have not addressed if MFAP4, apart from its direct haptotactic effects, can promote monocyte migration indirectly through upregulation of chemokine expression or integrin receptor availability. Finally, we did not investigate thrombus formation and biomechanical properties, which also contribute to AAA formation (54) and could be affected by the observed *Mfap4*-deficient phenotype.

In conclusion, our study provides evidence that MFAP4 deficiency alleviates macrophage accumulation and MMP production, leading to attenuated AAA formation. Even though contemporary interventions have considerably reduced the mortality of AAA (55, 56), the remaining high mortality rate warrants the search for new pharmacological approaches against AAA progression. Our findings strongly indicate that MFAP4 aggravates vascular inflammation and remodeling, suggesting that MFAP4 targeting may be a novel potential therapeutic avenue for vascular inflammatory diseases.

## Data Availability Statement

The raw data supporting the conclusions of this article will be made available by the authors, without undue reservation.

## Ethics Statement

The animal study was reviewed and approved by National Animal Experiments Inspectorate of Denmark.

## Author Contributions

AS, JS, G-PS, and GS conceived the study. BP, PC, KK-M, AS, KK, MD, NPM, SH, and CB performed the experiments and analyzed the data. JS, PH, TA, JM, NM, and VA participated in data analysis. BP, PC, KK-M, and GS wrote the manuscript. All authors revised and approved the manuscript.

## Funding

This work was supported by Novo Nordisk Fonden—project grants in clinical and translational medicine (22360), the Danish Research Council (7016-00038B), the Lundbeck Foundation (R164-2013-15355), Th. Maigaards eftf. Fru Lily Benthine Lunds Fond af 1.6.1978, Familien Hede Nielsens Fond, Snedkermester Sophus Jacobsen og Hustru Astrid Jacobsens Fond, Beckett Fonden, Fonden til Lægevidenskabens Fremme, Fondsbørsvekselerer Henry Hansen og Hustru Karla Hansen Født Vestergaards Legat, Frimodt-Heineke Fonden, and Torben og Alice Frimodts Fond.

## Conflict of Interest

AS and GS are inventors of patents owned by the University of Southern Denmark WO2014114298 and EP17199552.5. PH is employed by Astra Zeneca. The remaining authors declare that the research was conducted in the absence of any commercial or financial relationships that could be construed as a potential conflict of interest.

## Publisher's Note

All claims expressed in this article are solely those of the authors and do not necessarily represent those of their affiliated organizations, or those of the publisher, the editors and the reviewers. Any product that may be evaluated in this article, or claim that may be made by its manufacturer, is not guaranteed or endorsed by the publisher.
