# Supplementary material for: MFAP4 Deficiency Attenuates Angiotensin II-Induced Abdominal Aortic Aneurysm Formation Through Regulation of Macrophage Infiltration and Activity
Source: Front Cardiovasc Med. 2021 Nov 5;8:764337. doi: 10.3389/fcvm.2021.764337 (PMC8602692; doi:10.3389/fcvm.2021.764337)
Supplement: Supplementary file 1 [file Data_Sheet_1.PDF]

SUPPLEMENTARY MATERIAL

MFAP4 deficiency attenuates angiotensin II-induced abdominal aortic aneurysm formation through regulation of macrophage infiltration and activity

Bartosz Pilecki[1,†], Paulo V. S. D. de Carvalho[1,2,3,†], Katrine L. Kirketerp-Møller[1,†], Anders Schlosser[1], Karin Kejling[1], Magdalena Dubik[1], Nicklas P. Madsen[1], Jane Stubbe[4], Pernille B. L. Hansen[4,5], Thomas L. Andersen[6,7], Jesper B. Moeller[1,8], Niels Marcussen[6], Vasco Azevedo[2], Svend Hvidsten[9], Christina Baun[9], Guo-Ping Shi[10], Jes S. Lindholt[11], Grith L. Sorensen[1]

[1]Department of Cancer and Inflammation Research, Institute of Molecular Medicine, University of Southern Denmark, Odense, Denmark.

[2]Department of General Biology, Institute of Biological Sciences, Federal University of Minas Gerais (UFMG), Belo Horizonte, Brazil.

[3]Department of Mathematics and Informatics, University of Southern Denmark, Odense, Denmark.

[4]Department of Cardiovascular and Renal Research, Institute of Molecular Medicine, University of Southern Denmark, Odense, Denmark.

[5]Cardiovascular, Renal and Metabolism, IMED Biotech Unit, AstraZeneca, Gothenburg, Sweden.

[6]Department of Pathology, Odense University Hospital, Odense, Denmark.

[7]Pathology Research Unit, Institute of Clinical Research and Institute of Molecular Medicine, University of Southern Denmark, Odense, Denmark.

[8]Danish Institute for Advanced Study, University of Southern Denmark, Odense, Denmark.

[9]Department of Nuclear Medicine, Odense University Hospital, Odense, Denmark.

[10]Department of Medicine, Brigham and Women´s Hospital and Harvard Medical School, Boston, USA.

[11]Department of Thoracic, Heart and Vascular Surgery, Odense University Hospital, Odense, Denmark.

[†]These authors have contributed equally to this work and share first authorship.

Corresponding author: Bartosz Pilecki, Department of Cancer and Inflammation Research, Institute of Molecular Medicine, University of Southern Denmark, JB Winsloews Vej 25.3, 5000 Odense C, Denmark. E-mail: bpilecki@health.sdu.dk

Expanded Materials and Methods

Experimental animals

Animals were housed in conventional cages at the Biomedical Laboratory, Institute of Molecular Medicine, University of Southern Denmark and kept on a 12:12 hours light-dark cycle at a controlled temperature of 21 ± 1 ºC. Free access to water and either standard chow (Altromin no.1324, GmbH & Co. KG, Lage, Germany) or western diet (D12079B, RD Western Diet, Research Diets In., New Brunswick, USA) was provided.

Genotyping

Tail biopsies were obtained from 3-week-old mice, and DNA extraction was performed using the REDExtract-N-Amp^{TM} Tissue PCR Kit (Sigma-Aldrich, St. Louis, Mo, USA) according to the manufacturer's instructions. Multiplex PCR consisting of a forward primer 5´-GGCGGCTCTGAAACCAATTA-3´ and reverse primers 5´-CCCGGCCCAAAGGAGAAATCT-3´ and 5´-GCTCCAGACTGCCTTGGGAA-3´ (Sigma-Aldrich, St. Louis, MO, USA) was used to identify the wild-type allele and the *Mfap4*$^{-/-}$ allele, respectively. The *ApoE*$^{-/-}$, *ApoE*$^{+/-}$, *and ApoE*$^{+/+}$ genotypes were identified using a forward primer 5´-GCCTAGCCGAGGGAGAGCCG-3´ and reverse primers 5´-TGTGACTTGGGAGCTCTGCAGC-3´ and 5´-GCCGCCCCGACTGCATCT-3´ for the wild-type allele and *ApoE*$^{-/-}$ allele, respectively (Sigma-Aldrich, St. Louis, MO, USA).

Arterial blood pressure measurement

Arterial blood pressure was measured continuously from day 14 to day 21 after osmotic minipump installation in mice subjected to Ang II infusion. Ten days after pump installation the mice were anesthetized by an intraperitoneal injection of 100 mg/kg ketamin (Ketaminol Vet, MSD Animal Health, Ballerup, Denmark) and 10 mg/kg xylazine (Rompun Vet, Bayer, Health Care, healthcare.bayer.com). Catheters for blood pressure measurements were placed in the femoral artery and connected to a polyethylene catheter, which was tunnelled subcutaneously from the femoral artery to the neck of the mouse. The catheter was connected to a swivel (Instech Laboratories, Plymouth Meeting, PA, USA) allowing the mice to move freely in the cage. During the following days of recovery, catheter patency was maintained by infusions of 10 μl/h heparin solution (100 U/ml in glucose). Before initiation of blood pressure measurements (day 14), the arterial line was connected to a pressure transducer (Föhr Medical Instruments, Seeheim-Ober Beerbach, Germany), and data were collected at 200 Hz using Lab View software (National Instruments, Austin, TX, USA). Mean arterial blood pressure (MAP) and heart rate (HR) values were generated for every 5 minutes for seven days and calculated separately for the day and night periods.

Micro-CT assessment of vascular luminal volume

28 days after osmotic minipump installation the Ang II-induced vascular luminal volume was visualized using micro-CT imaging. Mice were anesthetized by subcutaneous injection of a solution of 1.26 μg/g body weight Hypnorm (Veta Pharma, Leeds, UK) 20 μg/g Dormicum (Midazolam, Hameln Pharmaceuticals, Gloucester, UK). Subsequently, 100 ul ExiTron nano12000 nanoparticulate contrast (Viscover™ ExiTron™ nano, Miltenyi Biotec, Lund, Sweden) was injected intravenously through the tail vein. Prior to micro-CT imaging, the mice were placed in a supine position in the micro-CAT II scanner (Siemens Inveon Standard, Siemens Pre-Clinical Solutions, Knoxville, TN, US). During 360° rotation, 720 projections were acquired with a source voltage of 80 kV and beam current of 500 μA. The magnification was set to a medium resolution, and the source to detector distance was 39.7 cm. The exposure time was 900 ms per projection. The total acquisition time was approximately 35 minutes. Using the Dose Calculator software (Siemens, Knoxville, TN, US), the absorbed radiation dose of the animal was estimated to be approximately 1.1Gy. Images were reconstructed with a filtered back projection Feldkamp algorithm and scaled to Hounsfield units (HUs) using acquisition software IAW version 1.5 (Siemens Pre-Clinical Solutions, Knoxville, TN, US). The reconstructed images had isotropic voxels of 39.4 μm in size and a matrix size of 1024x1024x1024. Mice were sacrificed immediately after the scanning procedure. The region of interest for assessment of the abdominal aorta lumen volume was set to cover vertebrae L1-L4. To segment the aorta lumen in this region the semiautomatic built-in random walk algorithm of the Siemens Research software was applied. To initiate the segmentation algorithm seed points within the aorta lumen and just outside the aorta were manually defined in a blinded manner.

Quantitative reverse transcription PCR (RT-qPCR)

Tissues for RNA purification were sampled from 12-week-old wild-type male C57BL/6N mice. Total RNA was extracted using TRIzol reagent (Life Technologies, Thermo Fisher, Waltham, MA, USA) and zirconium oxide beads (1.4 or 2.8 mm, Bertin Technologies, Paris, France) using a Precellys®24 homogenizer (Precellys®, Bertin Technologies, Paris, France) according to the manufacturers' recommendations. RNA concentration was measured using the NanoDrop spectrophotometer (Thermo Fisher).

250 ng purified RNA from each sample was used for cDNA synthesis using random hexamers (Applied Biosystems, Life Technologies, Thermo Fisher) and M-MLV Reverse Transcriptase (Sigma-Aldrich) according to the manufacturers' recommendations. TaqMan Universal Master Mix II (4440040, Applied Biosystems) and TaqMan Gene Expression Assays (Applied Biosystems, Life Technologies, Thermo Scientific, Waltham, MA, USA) were used for the following Taqman probes: *Mfap4* (Mm00840681_m1), *Col1a1* (Mm0080166_g1), and eucaryotic 18S rRNA (4333760F, endogenous control). Reactions were performed using a StepOnePlus Real-Time PCR system (Applied Biosystems, Life Technologies, Thermo Fisher). Relative mRNA levels of *Mfap4* and *Col1a1* were calculated using the $2^{\Delta Ct}$ method using qBase+ software (Biogazelle, Zwijnaarde, Belgium).

Serum lipid measurements

Serum total cholesterol and triglyceride concentrations were measured using commercially available enzymatic colorimetric assays (ABX Pentra Cholesterol CP and ABX Pentra Triglycerides CP, Horiba Ltd. Minami-ku Kyoto, Japan) according to the manufacturer's recommendations.

MMP zymography

Tissue homogenates were made from suprarenal aortic tissue from saline- and Ang II-infused mice 9 days after osmotic minipump implantation. Tissues were homogenized in 150 µl TBS containing 0.1% Triton X-100 (Sigma-Aldrich, St. Louis, MO, USA) and protease and phosphatase inhibitors (Mini cOmplete protease inhibitor cocktail tablet and PhosSTOP phosphatase inhibitor cocktail tablet, both from Roche, Basel, Switzerland). Zymography was performed to measure the activity of MMP-2 and MMP-9. Samples and markers (Proenzyme, Calbiochem, Merck Millipore, Darmstadt, Germany) normalized for protein concentration were loaded on 10% Novex® Zymogram Gelatin Gels (Novex®, Life Technologies, Thermo Scientific, Waltham, MA, USA) and the gels were developed according to the manufacturer's instructions. The MMP activity was visualized using the Fusion x7 (Vilber Lourmat France) and the FUSION-CAPT version 15.18 software (Science Imaging Scandinavia AB, Saltsjöboo, Sweden). Band intensity was analyzed using GelQuant.NET software (biochemlabsolution.com), and MMP activity was shown as intensity relative to an inter-gel marker control.

Expression of recombinant MFAP4

ExpiCHO-S cells were transfected with a human MFAP4 expression plasmid (pcDNA 3.4-hMFAP4 TOPO® TA, Thermo Fisher) according to the manufacturer's instructions. After adding ExpiFectamine CHO Enhancer and ExpiCHO Feed, the cells were incubated at 37°C in 8% CO_2 for 10 days with shaking. Recombinant MFAP4 protein released into the cell supernatant was purified by affinity purification as described previously[21] followed by anion ion-exchange chromatography on a Resource Q column (GE Healthcare Life Sciences) on a Äkta FPLC apparatus (Amersham Pharmacia Biotech). The purity of rMFAP4 was tested by SDS-PAGE followed by Coomassie staining using SimplyBlueTM SafeStain (Invitrogen). Potential endotoxin contamination was assessed using the Limulus amebocyte lysate assay (Lonza); the endotoxin levels were below 2 EU/mg protein.

Supplemental Figures and Figure Legends

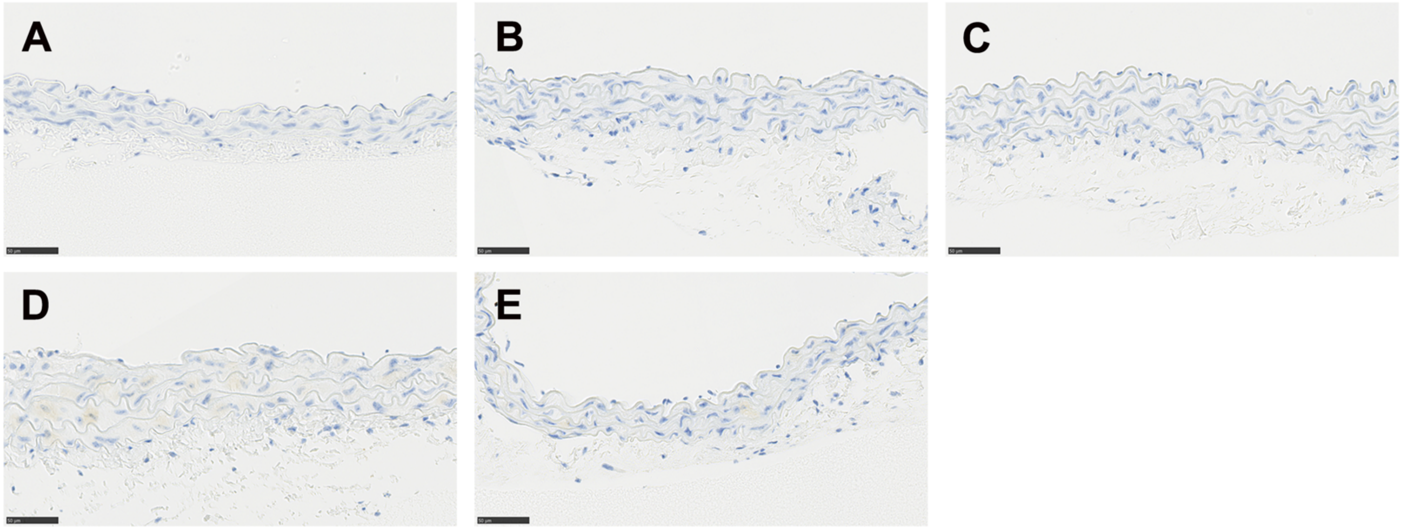

Supplementary Figure 1. Representative isotype control stainings. (A) Rabbit IgG. **(B)** Mouse IgG2a-FITC. **(C)** Rat IgG2a. **(D)** Rat IgG2b. **(E)** Goat IgG. Scale bar = 50 μm.

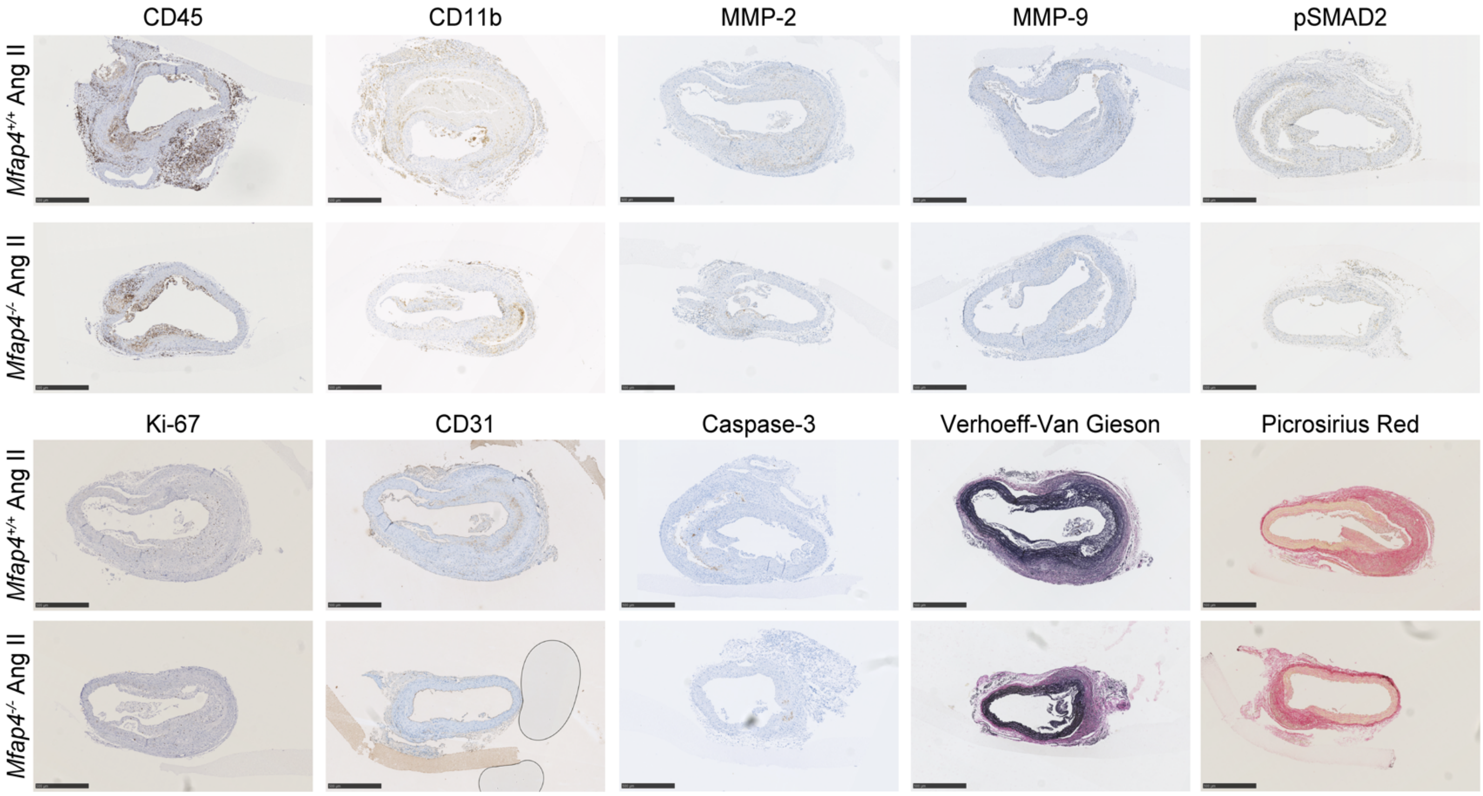

Supplementary Figure 2. Representative stainings of entire aortic sections of *ApoE$^{-/-}$* (*Mfap4$^{+/+}$*) and *ApoE$^{-/-}$ Mfap4$^{-/-}$* (*Mfap4$^{-/-}$*) mice. Scale bar = 500 μm.

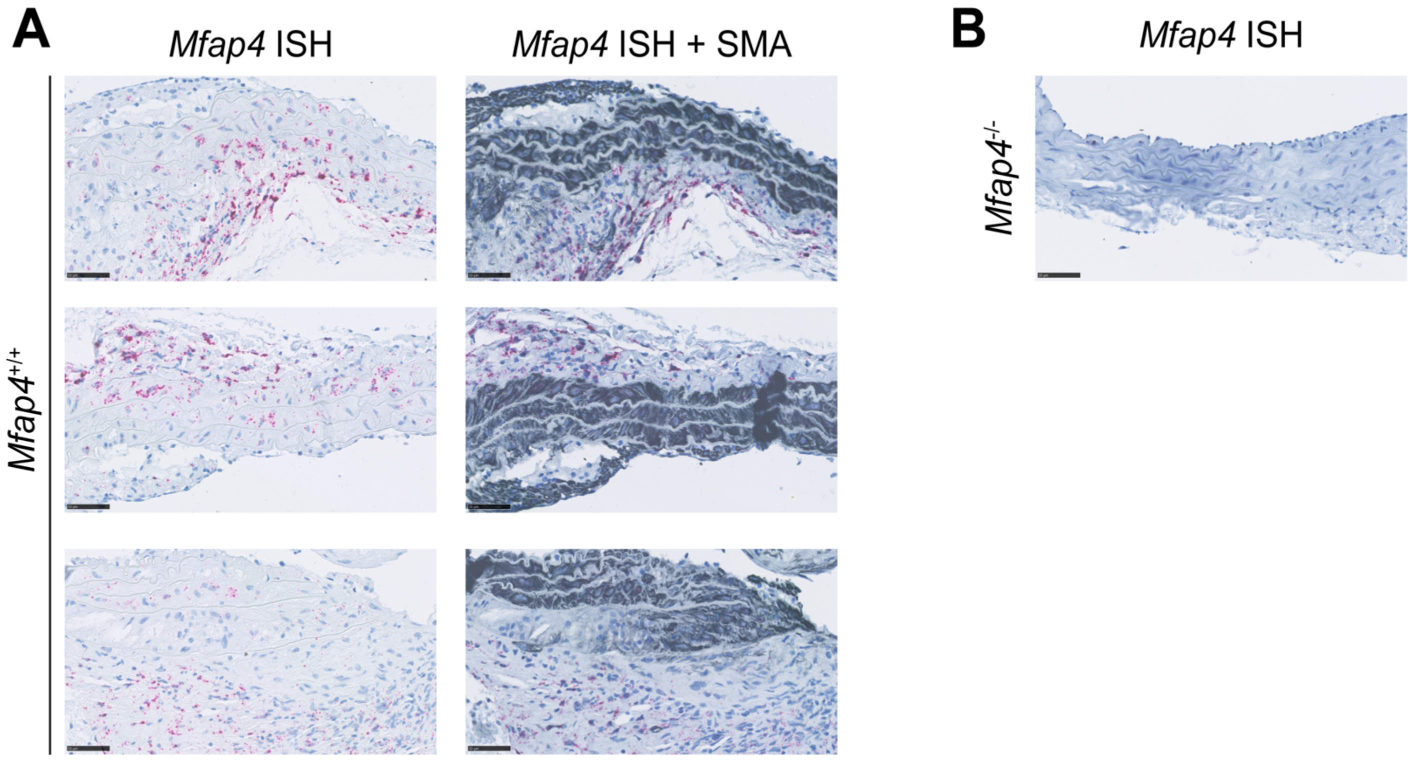

Supplementary Figure 3. ***Mfap4* in situ hybridization (ISH) staining. (A)** Examples of representative *Mfap4* ISH staining (pink) of *ApoE*$^{-/-}$ (*Mfap4*$^{+/+}$) aortas together with α-smooth muscle actin (SMA; black) co-staining. *Mfap4* is expressed in both SMA-positive and SMA-negative adventitial cells as well as medial SMCs. **(B)** Control ISH staining of *ApoE*$^{-/-}$ *Mfap4*$^{-/-}$ (*Mfap4*$^{-/-}$) aorta.

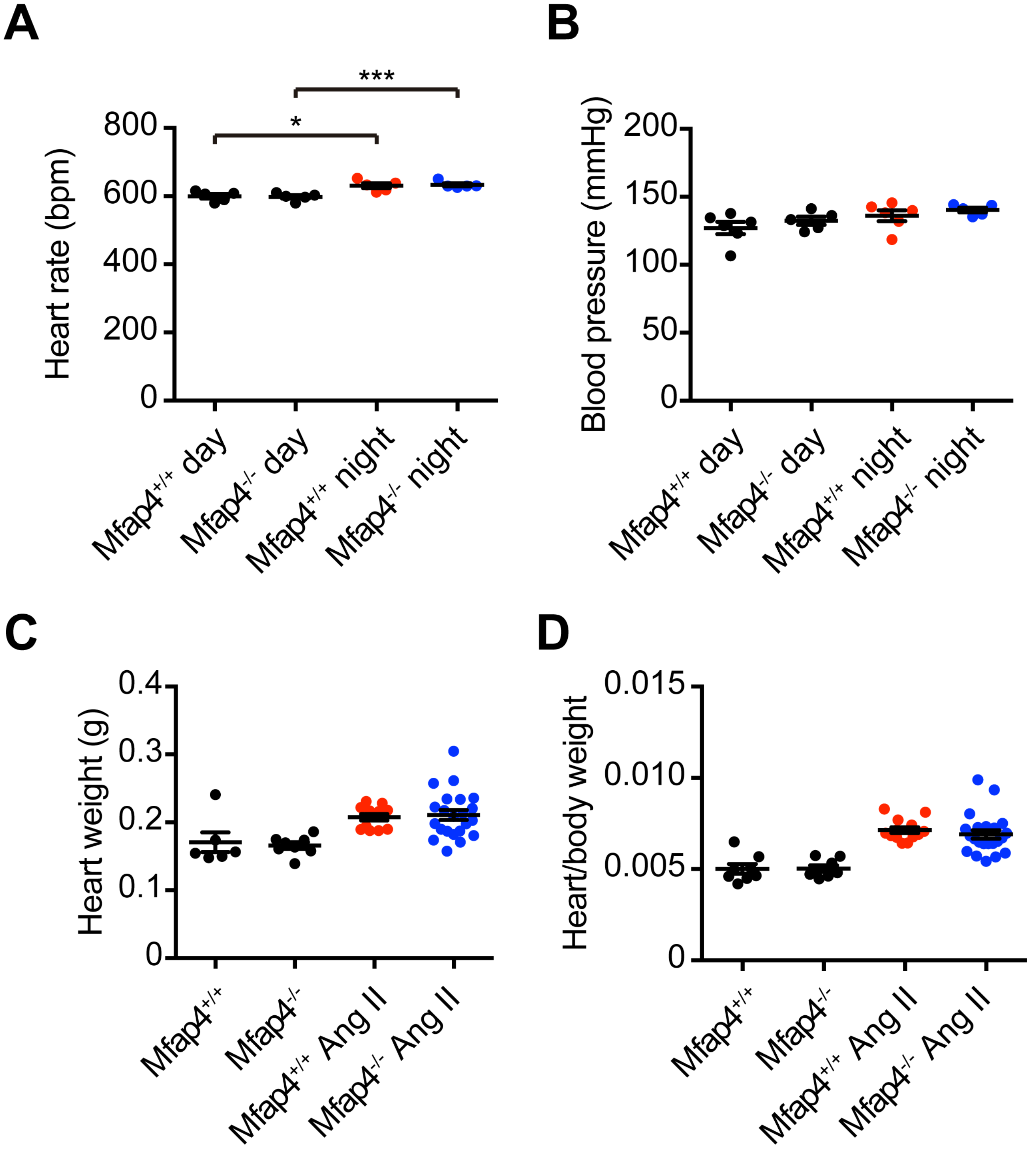

Supplementary Figure 4. Heart rate, mean arterial blood pressure and heart weight development after Ang II infusion in *ApoE*$^{-/-}$ mice are independent of *Mfap4* genotype. (A) Heart rate and **(B)** mean arterial blood pressure were measured continuously for 7 days (day and night) at the interval from day 14 to 21 during saline or Ang II infusion. n=5-6. **(C)** Heart weight after 28 days. **(D)** Heart-to-body weight ratio measured after 28 days. n=6-8 (saline), 12-22 (Ang II). *$p<0.05$, ***$p<0.001$, analyzed with Mann-Whitney U test. bpm, beats per minute.

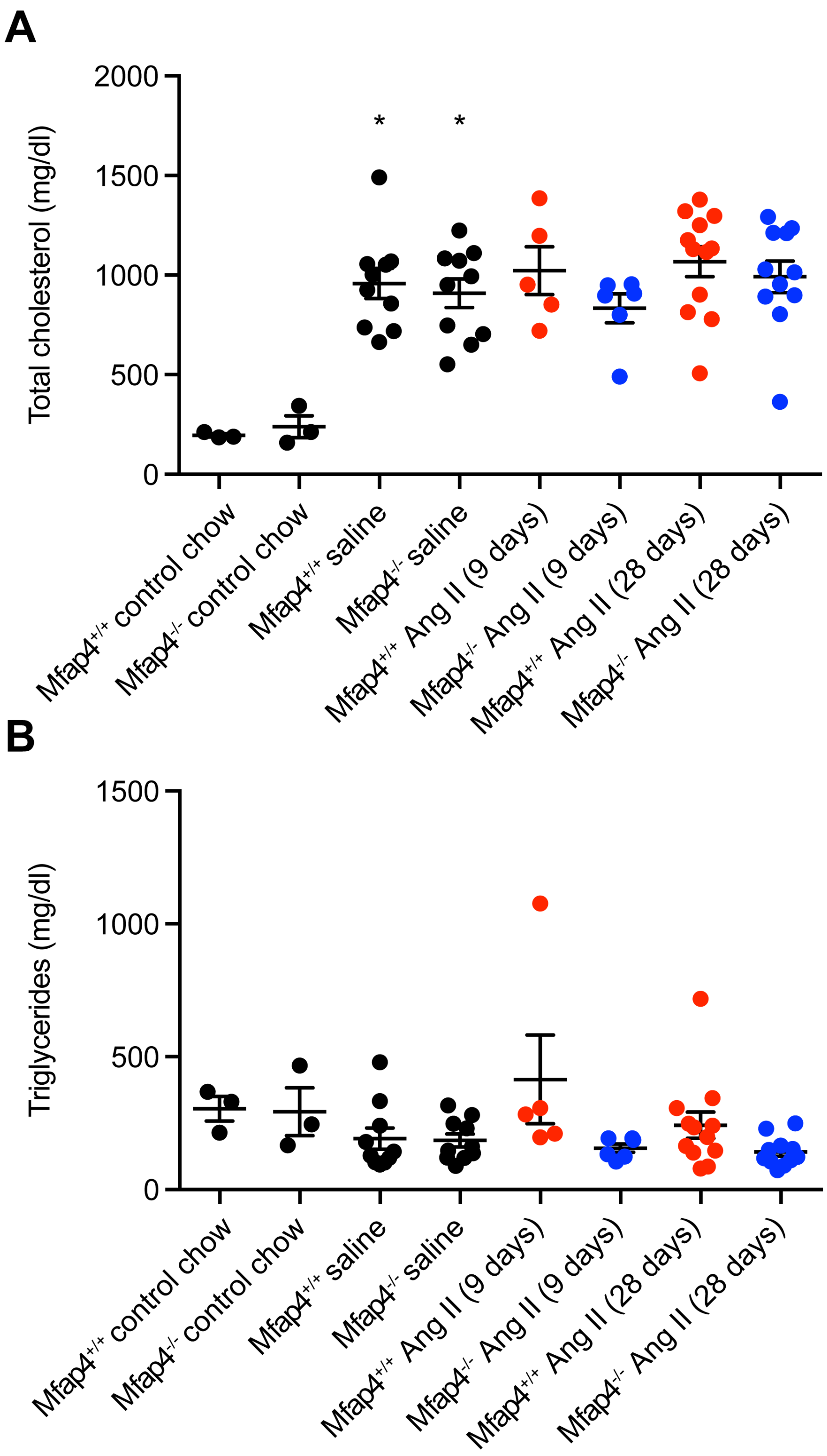

Supplementary Figure 5. Total serum cholesterol and triglyceride levels are independent of *Mfap4* genotype. Total cholesterol and triglyceride concentrations were measured in serum from control chow-fed *ApoE*$^{-/-}$ (*Mfap4*$^{+/+}$) and *ApoE*$^{-/-}$ *Mfap4*$^{-/-}$ (*Mfap4*$^{-/-}$) mice and western diet-fed *Mfap4*$^{+/+}$ and *Mfap4*$^{-/-}$ mice infused with saline or Ang II for 9 or 28 days. **(A)** Total serum cholesterol levels. **(B)** Total serum triglyceride levels. n=3-12. *p<0.05 between control chow-fed and western diet-fed mice, analyzed with Mann-Whitney U test.

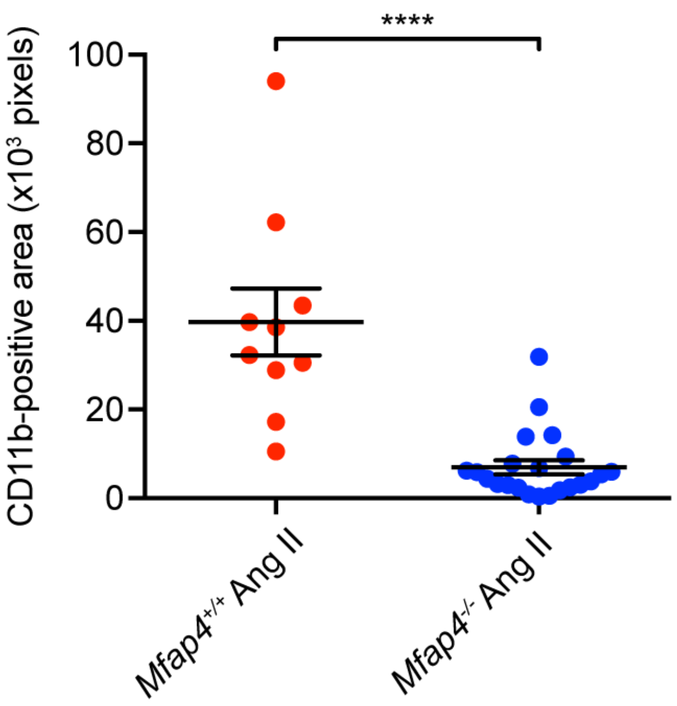

Supplementary Figure 6. MFAP4 deficiency reduces CD11b-positive cell infiltration within the adventital layer of Ang II-infused AAAs. Morphometric analysis of adventitial CD11b staining of aortic sections of *ApoE*$^{-/-}$ (*Mfap4*$^{+/+}$) and *ApoE*$^{-/-}$*Mfap4*$^{-/-}$ (*Mfap4*$^{-/-}$) mice after 28 days of Ang II infusion. n=10 (*Mfap4*$^{+/+}$ Ang II), 22 (*Mfap4*$^{-/-}$ Ang II).****p<0.0001, analyzed with Mann-Whitney U test.

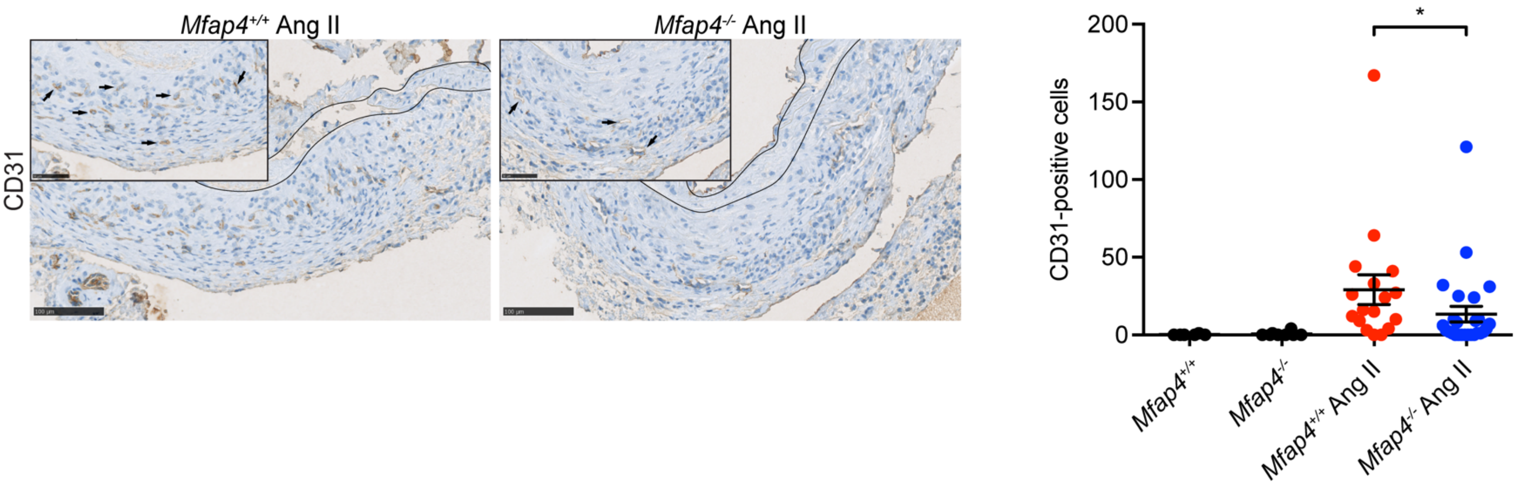

Supplementary Figure 7. MFAP4 deficiency reduces the microvessel number in Ang II-infused AAAs. Morphometric analysis of CD31 stainings of aortic sections of *ApoE*$^{-/-}$ (*Mfap4*$^{+/+}$) and *ApoE*$^{-/-}$*Mfap4*$^{-/-}$ (*Mfap4*$^{-/-}$) mice after 28 days of Ang II infusion. Black arrows indicate examples of positive cells. Black lines delineate borders between intimal, medial and adventitial layers. n=6-8 (saline), 15-26 (Ang II). Representative pictures are shown. Scale bar = 100 µm/50 µm. *p<0.05, analyzed with Mann-Whitney U test.

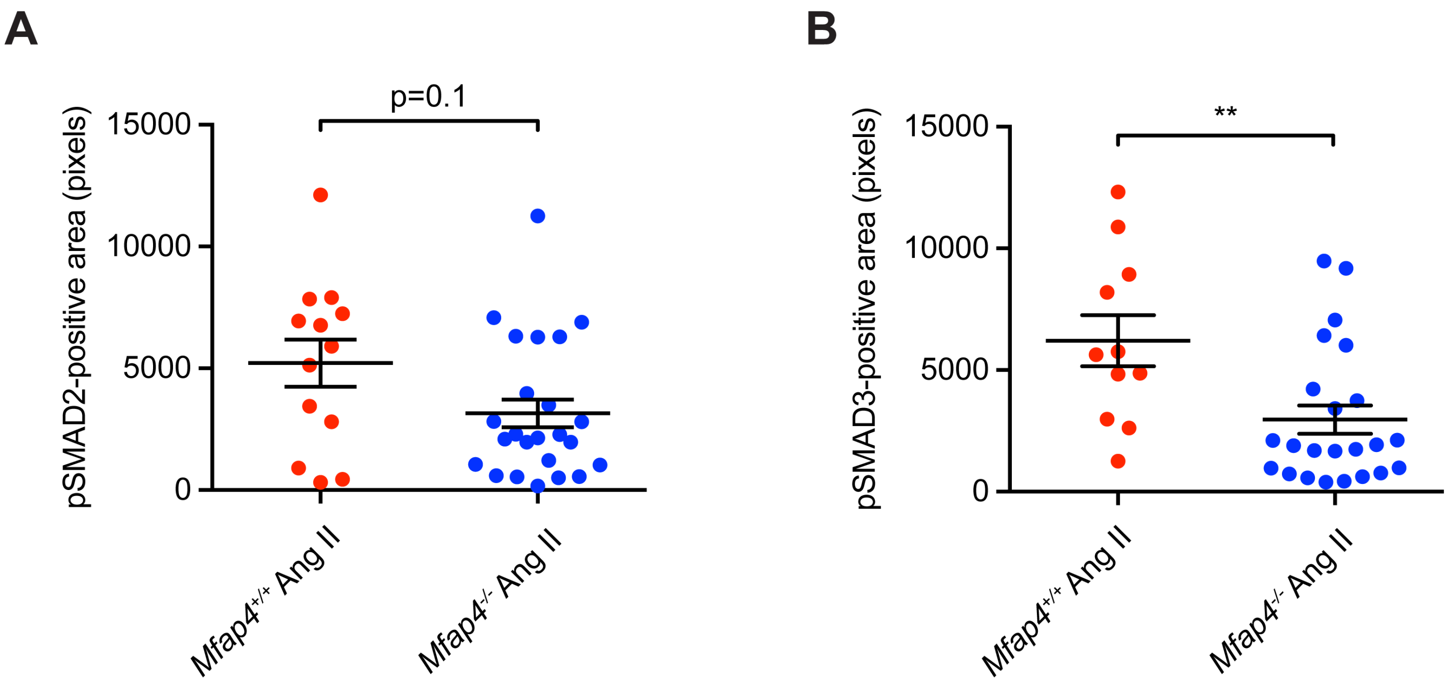

Supplementary Figure 8. MFAP4 deficiency reduces SMAD phosphorylation in the medial layer of Ang II-infused AAAs. Morphometric analysis of pSMAD2 (A) and pSMAD3 (B) stainings of aortic sections of *ApoE*$^{-/-}$ (*Mfap4*$^{+/+}$) and *ApoE*$^{-/-}$*Mfap4*$^{-/-}$ (*Mfap4*$^{-/-}$) mice after 28 days of Ang II infusion. n=11 (*Mfap4*$^{+/+}$ Ang II), 23-24 (*Mfap4*$^{-/-}$ Ang II). **p<0.01, analyzed with Mann-Whitney U test.

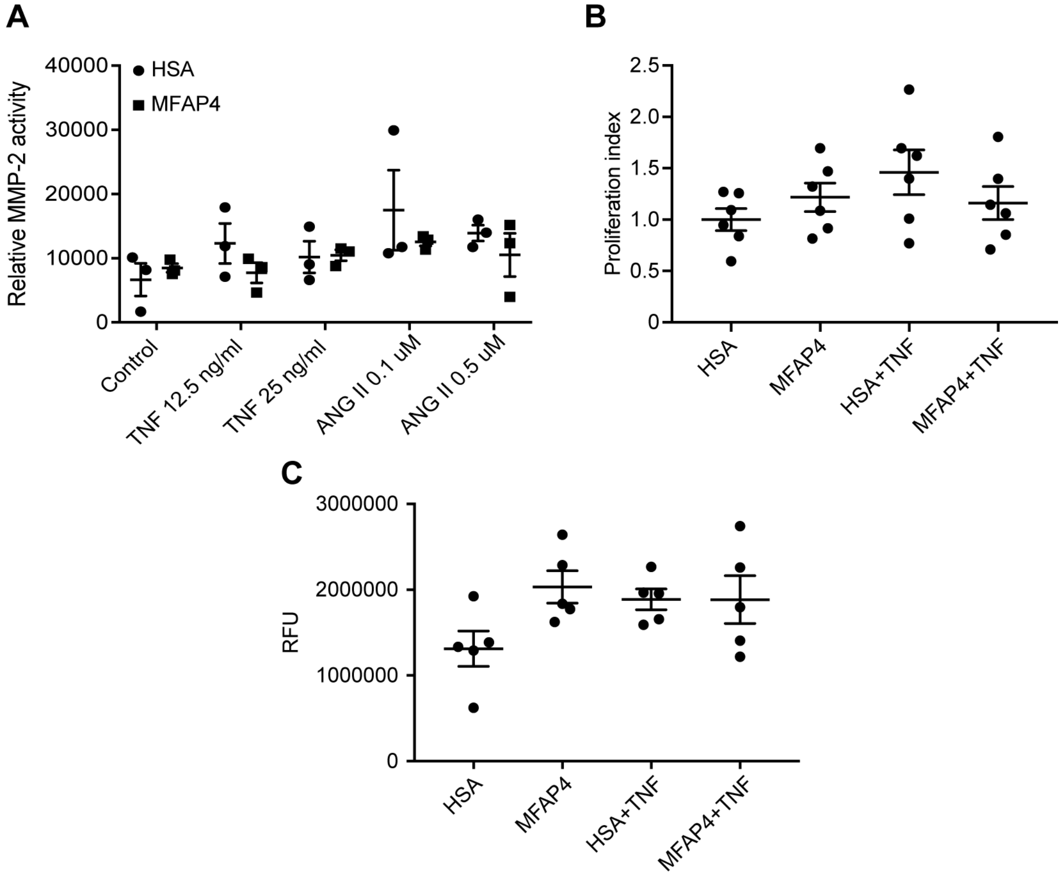

Supplementary Figure 9. MFAP4 stimulation does not influence MMP-2 activity in smooth muscle cells or proliferation and viability of macrophage-like cells. (A) Relative MMP-2 activity of fetal human aortic smooth muscle cells seeded on human serum albumin (HSA) or immobilized MFAP4 together with TNF or Ang II co-stimulation after 24 h is shown. **(B-C)** Proliferation (B) and viability (C) of differentiated and TNF-stimulated THP-1 macrophage-like cells is not significantly affected by MFAP4 co-stimulation. of n=3-6 independent experiments. Data were analyzed with one-way ANOVA followed by Bonferroni's test or Mann-Whitney U test.

Supplementary Tables

Target antigen	Vendor or Source	Catalog #	Working concentration	Clone	Isotype
α-SMA-FITC	Sigma	F3777	2 μg/ml	1A4	Mouse IgG2a-FITC
Cleaved caspase-3	Cell Signaling Technology	9661	0,519 μg/ml	polyclonal	Rabbit IgG
CD45	BD Biosciences	550539	2,5 μg/ml	30-F11	Rat IgG2b
F4/80	AbD Serotec, Bio-Rad	MCAP497	10 μg/ml	CI:A3-1	Rat IgG2b
Ki67	Dako	M7249	1:100	TEC-3	Rat IgG2a
MMP-9	Abcam	ab38898	10 μg/ml	polyclonal	Rabbit IgG
MMP-2	Abcam	ab37150	1 μg/ml	polyclonal	Rabbit IgG
CD31	Santa Cruz Biotechnology	sc1506	1 μg/ml	polyclonal	Goat IgG
pFAK	Abcam	ab4803	1:5000	polyclonal	Rabbit IgG
CD11b	Abcam	ab133357	0,3375 μg/ml	EPR1344	Rabbit IgG
pSMAD2	Invitrogen	44-244G	0,625 μg/ml	polyclonal	Rabbit IgG
MFAP4-FITC	In-house	-	1:100	HG-HYB 7-14	Mouse IgG1-FITC
Rabbit IgG	Thermofisher	31235	Isotype control	-	Rabbit IgG
Mouse IgG2a-FITC	Thermofisher	11-4724-81	Isotype control	eBM2a	Mouse IgG2a-FITC
Rat IgG2a	Thermofisher	14-4321-85	Isotype control	eBR2a	Rat IgG2a
Rat IgG2b	Thermofisher	14-4031-85	Isotype control	eB149/10H5	Rat IgG2b
Goat IgG	Thermofisher	02-6202	Isotype control	-	Goat IgG
Ki67	Thermofisher	MA5-14520	0,058 μg/ml	SP6	Rabbit IgG
CD45-PE	Biolegend	103106	2,67 μg/ml	30-F11	Rat IgG2b
α-SMA-Cy3	Sigma	C6198	5 μg/ml	1A4	Mouse IgG2a
Rabbit IgG-Alexa Fluor Plus 647	Thermofisher	A32733	4 μg/ml	-	Goat IgG

Supplementary Table 1. Lis t of antibodies.
